# Heterogeneous Heat Absorption Is Complementary to Radiotherapy

**DOI:** 10.3390/cancers14040901

**Published:** 2022-02-11

**Authors:** Andras Szasz

**Affiliations:** Biotechnics Department, Szent Istvan University, H-2040 Budaors, Hungary; Szasz.Andras@gek.szie.hu

**Keywords:** loco-regional hyperthermia, oncology, modulated electro-hyperthermia, cellular selection, bioelectromagnetics, complexity, immune-effects

## Abstract

**Simple Summary:**

This review shows the advantages of heterogeneous heating of selected malignant cells in harmonic synergy with radiotherapy. The main clinical achievement of this complementary therapy is its extreme safety and minimal adverse effects. Combining the two methods opens a bright perspective, transforming the local radiotherapy to the antitumoral impact on the whole body, destroying the distant metastases by “teaching” the immune system about the overall danger of malignancy.

**Abstract:**

(1) Background: Hyperthermia in oncology conventionally seeks the homogeneous heating of the tumor mass. The expected isothermal condition is the basis of the dose calculation in clinical practice. My objective is to study and apply a heterogenic temperature pattern during the heating process and show how it supports radiotherapy. (2) Methods: The targeted tissue’s natural electric and thermal heterogeneity is used for the selective heating of the cancer cells. The amplitude-modulated radiofrequency current focuses the energy absorption on the membrane rafts of the malignant cells. The energy partly “nonthermally” excites and partly heats the absorbing protein complexes. (3) Results: The excitation of the transmembrane proteins induces an extrinsic caspase-dependent apoptotic pathway, while the heat stress promotes the intrinsic caspase-dependent and independent apoptotic signals generated by mitochondria. The molecular changes synergize the method with radiotherapy and promote the abscopal effect. The mild average temperature (39–41 °C) intensifies the blood flow for promoting oxygenation in combination with radiotherapy. The preclinical experiences verify, and the clinical studies validate the method. (4) Conclusions: The heterogenic, molecular targeting has similarities with DNA strand-breaking in radiotherapy. The controlled energy absorption allows using a similar energy dose to radiotherapy (J/kg). The two therapies are synergistically combined.

## 1. Introduction

Nowadays, oncology is one of the most complex interdisciplinary experimental and clinical research fields. Clinical success often relies on the sensitive balance between cure and toxicity, providing the most effective but at the same time the safest treatment. Hyperthermia (HT) has promised a simple way to solve the frequent dilemma of complementary treatment choice. Despite its promise and a long history with ancient roots, oncological hyperthermia has had a long and bumpy road to modern medicine, and even today, it has no complete acceptance among oncology professionals. The original ancient idea of hyperthermia is relatively simple: heat the tumor, which forces it to use more resources from the host tissue due to accelerated metabolism, but no extra supply is available. The “starving” tumor destroys itself by acidosis. A deep belief in the curative effect of the fever-like processes, which force self-control of the body, drives the medical concept of “Give me the power to produce fever and I will cure all diseases” [1]. Hippocrates successfully applied radiative heat to treat breast cancer [1]. In vitro measurements have proved this idea [2], measuring a significant impoverishment of Adenosine triphosphate (ATP) and lactate enrichment in treated tumors.

**Figure 1:**
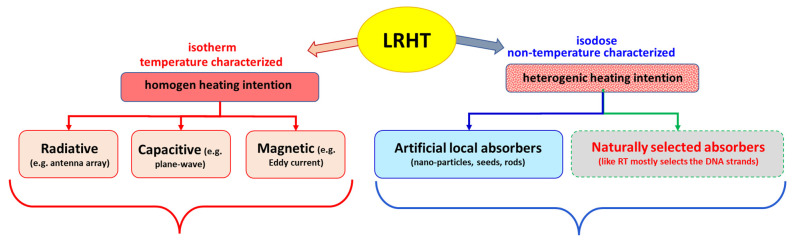
The two essential branches of electromagnetic LRHT methods. The majority of applications use the conventional focusing with isothermal intention. The method requests to measure the temperature as dose characterization. Heterogeneous (non-isothermal) heating is an emerging category of LRHT applications with nanoparticle insertion (mainly magnetic suspension). The heterogenic heating methods do not need direct temperature measurement. The dose measures the absorbed energy (J/kg = Ws/kg), so the tumor’s temperature develops by the heat-conduction from the targeted particles. The figure does not show the popular non-electromagnetic LRHT methods (e.g., HIPEC and HiFu).

The large group of HT methods contains various therapies using various electromagnetic and mechanical (ultrasound) energy sources. The attention of hyperthermic oncology presently focuses on local-regional heating (LRHT) methods by electromagnetic effects. There are two basic categories of LRHT heating; Figure 1.

1. External radiation focused on the target, trying to heat the tumor mass as homogeneously as possible without considerably heating surroundings tissues. The heating intention is isothermal, but due to the heterogeneity of the target and the heat distribution dynamics controlled by blood flow, the temperature is not homogeneous (see later). The intensive heating of a larger volume (regional heating) achieves an approximately controllable condition in the tumor at the central position. The treatment evaluation involves the ratio of the isothermal areas. The specific power density (SAR) ranges from 4.6 to 89 W/kg [3], depending on the location and size of the tumor, determining the heated volume and its blood flow.
2. Heating good energy absorbers in a localized area by electromagnetic effects, which heats these materials extensively, and in the next step, the absorbers heat up their host tissues. The heating intention is heterogeneous, targets only the dedicated particles (like nanoparticles, seeds, rods, etc.). The dose homogeneity characterizes this method because of the dispersed absorbers. The particles heat up their environment by heat-conduction, realizing more localized heating in the volume. The SAR in nanoparticle methods is surprisingly large because the absorbers have only a tiny mass compared to the surrounding tissue. The small mass (ranging density of 1 $\mathrm{mg}/{\mathrm{cm}}^{3}$ specifically absorbs extra-large SAR >> 1 W/g = 1 kW/kg or higher [4], because of the absorption on the tiny target. When it heats the neighboring tissues, the average SAR corresponds to the isothermal heating conditions in the range of about a few W/kg. Targeting various chemical bonds uses even higher SAR because the absorbing mass is lighter than the metallic nanoparticle. These methods focus on molecular changes. The temperature is a possible cofactor.

The success of LRHT is unquestionably conclusive. Results regarding many tumors, including breast [5], head and neck [6], cervix [7], pancreas [8], soft-tissue sarcoma [9], and others [10], provide convincing proof of its place in the field of oncotherapies. In particular, LRHT has had remarkable success, such as in a complementary application with radiation therapy (RT) [11,12,13,14], showing a solid synergy [15,16] and being applied successfully in various curative therapies [17,18,19]. The success of complementary RT + LRHT has a broad spectrum of clinical evidence [20,21,22,23,24], and has been well-reviewed in its details [25,26,27,28]. The introduced thermal enhancement ratio (TER) characterizes LRHT’s additional gain over RT [29].

Together with the high rate of successes, challenges, of course, also appear. To fulfill our strong motivation to popularize LRHT among oncology professionals, we analyze some of the apparent controversies in LRHT applications, studying these challenges in the search for a solution. The challenges are not limiting but oppositely motivate us to solve the actual difficulties and thereby seize the extreme medical value of hyperthermia in oncology. The challenge guides us to new developments and improvements in the otherwise broad spectrum of hyperthermia facilities in oncology.

### 1.1. Heating Challenge

The skeptical opinion concerning hyperthermia in oncology was developed in parallel with expectations. A half-century ago, in 1964, a leading German oncosurgeon expressed his doubts [30]: “All of these methods impress the patient very much; they do not impress their cancer at all”. His skepticism towards oncological hyperthermia became widespread among medical experts, who declared hyperthermia to be of no benefit to cancer patients and so did not propose it enter actual therapy protocols. Unfortunately, the method has to fight hard for its well-deserved place among stable routine therapies in oncology. Our task is to show the place of HT as the regular fourth column in the oncology arsenal, together with surgery, chemo, and radiotherapies.

The challenges always concern the complex behavior of living organisms, which balances multiple oppositional regulatory feedbacks. The balance gives a character a “double-edged sword”, which determines a window of positive actions. When applied outside this window, the helpful actions act oppositely, the difference between support or degradation being only the dose.

The primary challenge connects hyperthermia to the standard systemic homeostatic thermal control according to the complexity. The body temperature provides fundamental conditions of the proper physiologic and molecular processes, so its stability is essential and ranges in a narrow 7/273 (~2.6%) interval in humans. The homeostatic control regulates the system, keeping it stable and adaptable. Heating locally or systemically attacks the regulatory stability, igniting non-linear physiological reactions to correct the system [31]. The body’s homeostatic control monitors thermal conditions and regulates its temperature and parts compared to a set-point in the hypothalamus [32], trying to re-establish the unheated temperature. The feedback regulation non-linearly increases the blood-flow (BF) [33,34], as an effective heat exchanger, as well as the regulation intensifying other physiological mechanisms to control conditions [35]. The reactive BF change causes most of the challenges in LRHT applications.

On the other hand, the reaction to the growing temperature also has a supporting behavior. It induces relatively significant protective heat shock proteins (HSPs) in the targeted cells. The extra stress by heating increases the HSPs only slightly in the otherwise heavily stressed malignant cells but causes a drastic gain (8–10 times) in the healthy ones [36]. The difference makes the malignant cells more vulnerable to the temperature increase than the well adapting healthy cells.

### 1.2. Complementary Challenge

The correct dose application of LRHT is a critical issue in the future of hyperthermia in oncology [37]. Furthermore, the complementary therapy of LRHT and RT requires the precise dosing of both components to ensure safe and reproducible effectivity. RT has a traditional, well-applicable, accepted dose, which determines the isodose by the equal energy absorption in Gy ($=\frac{J}{kg}$) in the chosen target. The isodose energy absorption is not directly dependent on the size of the tumor. The dose is homogeneously distributed across the entire tumor volume, independently of its size; the same dose is maintained in all volume units. The treatment defines the isodose (e.g., fractional dose for daily application) equally, and the complete sum of fractions composes the final dose, which depends on the tumor conditions (localization, size, stage, conditions, cellular specialties, etc.). It is fixed through the planning process and the focusing adjustments realized.

LRHT uses the temperature as an active part of the treatment, applying it for dose characterization. Contrarily, RT regards it as an adverse effect, causing burns and fibrotic conditions [38,39]. A fundamental difference between RT and LRHT appears in their treatment length, and consequently, the applied energies. RT applies a short shot with only a negligible effect on the physiological regulation, while the LRHT treatment time is long (usually 60 min), so homeostatic control is activated. The radiation focus also shows significant differences: the heating produced with LRHT spreads into non-targeted volumes in conductive and convective ways, while RT remains local, being well focused on the planned volume. The frequency of the standard treatments differs too: while fractional RT treats daily, LRHT, due to the HSP protection that develops, cannot be applied so frequently, requiring at least a 48 h break between applications. Unfortunately, the LRHT-produced HSP could be associated with radioresistance too, but on the other hand, LRHT influences numerous other molecular parameters which could sensitize to the RT [40].

RT and LRHT achieve therapeutic synergy in their complementary application despite the differences. The LRHT supports the RT by the thermosensitizing [41] and oxygenation of the target [42]. The active arrest of the cell cycle can realize an essential synergy in different phases by the RT and LRHT. RT is most active in the mitosis phase, while moderate heat shock arrests G1/S and G2/M cell-cycle checkpoints [43]. The LRHT predominantly acts in the S phase of the cell cycle [44] in moderately acidic, hypoxic regions, complementing the cell cycle arrest. Various molecular parameters support the RT efficacy [45], e.g., a heat-induced decrease in DNA-dependent protein kinase [46].

The physiological regulation compensates for the heating effect of LRHT, increasing the BF by vasodilatation to maintain thermal homeostasis. The BF counterbalances the increased temperature by intensive heat-interchange, which in exchange delivers an extended oxygen supply for radio-effects, fixing the DNA breaks [47,48].

The possible synergy of RT and LRHT has a contradictory process. The high BF naturally opposes the Hippocratic “thermal starvation” concept. Nevertheless, the higher metabolic rate of the proliferating mass compensates for the missing supply by non-linearly increasing BF [49,50,51]. The effects of higher radiosensitivity compete with the increased volume of delivered nutrients due to vasodilation and the heat-promoted perfusion through the vessel walls. On the other hand, the neo-angiogenic arteries do not vasodilate in massive tumors, as they lack musculature in their vessel-wall [52].

Consequently, the reaction to heat differs in the healthy and malignant tissues, exhibiting approximately 38 °C when the BF in the tumor lags the BF in the healthy host [53]. Additionally, the temperature increase can produce vasoconstriction in certain tumors, which decreases the BF and the decrease in heat exchange offers a relatively higher temperature in these regions [54]. This effective heat trap [55] lowers the available oxygen, affecting the efficacy of RT. Parallel at the same time, vasodilatation in healthy tissues increases the relative BF, presenting more cooling media in the volume [56,57], and increases the RT effect in the healthy host tissue counterproductively to clinical safety.

The BF has a central role in maintaining the overall homeostasis. Besides the temperature, it regulates essential parameters like the acid-alkaline equilibrium, glucose delivery, immune actions, and numerous blood-delivered molecular feedback loops in the body. In the precise interaction of RT with LRHT, these parameters may also have remarkable modifying factors. The vascular response of tissues has a tumor-specific temperature threshold, indicated by the kink in the Arrhenius empirical plot [58,59], in consequence of a structural phase transition in the plasma membrane [60].

The above contradictory processes are natural in complex systems, where the suppressor–promoter pairs have an essential role in the dynamic regulation of the homeostatic balance. As always, the regulative processes balance the progressor and suppressor action, so not surprisingly, the radiotherapy-induced damage could cause the activation of damage-repair mechanisms, and survival signaling adds to other factors of tumor-resistive effects [61]. This complex dynamic behavior otherwise guarantees the robust stability of homeostasis as the regulator of healthy processes.

The complementary LRHT and RT synergy also require consideration of the system’s complexity. The sum of its distinct parts does not describe the natural cooperating procedures. The interactions are essentially nonlinear, representing that the whole is more than the sum of the parts. The living structures, in their complexity, have a universal behavior: they are self-organized [62]. The basic synergistic possibilities of LRHT and RT are collected in Table 1.

### 1.3. Dosing Challenge

**Figure 2:**
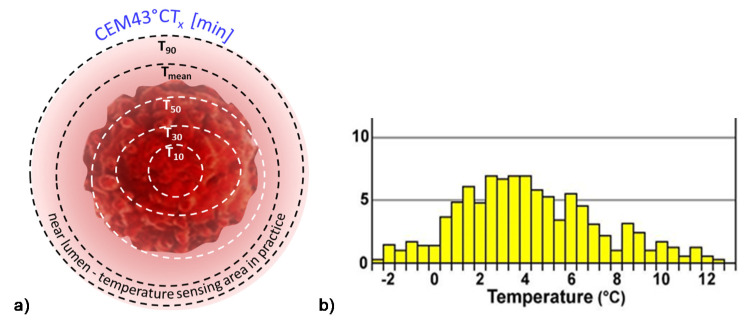
The heated focus rapidly spreads, so the temperature increases in a broader region. (**a**) The CEM43 dose depends on the isothermal areas, which differ by distance and develop by time. (**b**) The temperature distribution across the tumor after 64 min of treatment was measured by MRI (Pat.10. relapsed rectum carcinoma) [66].

The present dose of HT measured with cumulative equivalent minutes compared to the 43 °C basepoint, (CEM 43 ℃) [63,64] fit to the complete necrotic cell killing in vitro [65]. This reference is far from the reality of human medicine. The principal challenge of this dose is that homogenous heating is only an illusion. The approximately isothermal x percent of the heated area at T temperature completes the correct dose. The $CEM\;43\;℃\;T_{x}$ [65], where $T_{x}$ refers to the x% of the heated mass is approximated with the isothermal condition at temperature T. The dose is, of course, lowered by the growing x value; Figure 2. The isothermal approach tries macroscopically equalizing the temperature with high SAR. The $T_{x}$ estimation makes macro characterization and does not consider the tissue-defining microheterogeneity of the target.

The dosing of LRHT has serious challenges. It is much less reproducible and controllable than the dosing in RT. LRHT has huge anatomical, physiological, bio-electromagnetic, mechanical, and thermal heterogeneities, limiting the isodose-type approach of LRHT. The associated isothermal heating uses the temperature as a defining factor of the dose. However, the homogeneity and the lengthy treatment time do not maintain the otherwise precise focus. When the temperature stabilizes in a tiny region, the heat spreads from the targeted volume, and in this way, the intended isothermal region represents only a decreasing fraction of the target. The temporarily defined homogeneous volume may dynamically change by elapsed time; the situation is far from equilibrium [67], and the temperature and space distribution vary. The nonlinear BF and other homeostatic regulatory effects, together with the regular heat flow, destroy the homogeneity.

For example, when the measured temperature is actually $T_{90}$ in 90% of the monitored sites (referred to as the thermal isoeffect dose in 90% of the area), considering the average (assumed homogenous) volume, the $T_{90}>T_{80}>\ldots >T_{10}$, and the $T_{100}$ could be achieved only in a WBH situation. This construction certainly contradicts the homogenous idea.

Due to technical and safety issues in clinical conditions, achieving the 43 °C temperature requires enormous efforts. The challenge is heating the surrounding healthy host by the spread of heat that cannot be avoided with any precise focusing of the radiation beam. Clinical safety requests that the heating not exceed 42 °C in the healthy tissue. The blood flow increases more in the healthy host tissues than in the tumor, causing a particular gradient of the flow intensity to heat the tumor’s boundary. The tumor periphery contains the most vivid, mostly proliferative malignant cells. The temperature differences at the tumor border develop a certain BF gradient, which could wash out the aggressive malignant cells, increasing the risk of dissemination.

The $CEM\;43T_{x}$ dose has numerous principal challenges [68]. It failed to show the local control characterization of clinical results in soft tissue sarcomas [69] and does not correlate with clinical results for superficial tumors [70]. Complete homogeneity in the heating of living objects could be achieved only in the whole-body hyperthermia (WBH) process. It represents an entirely isothermal $CEM\;43\;℃\;T_{100}$ situation. Contrary to isothermal heating, the non-isothermal LRHT shows better clinical results [71], and the results of complementary application to chemotherapy also remain behind the chemotherapy alone [72,73]. However, administering a dose of $CEM\;43T_{90}$ LRHT also did not show a correlation between dose and clinical outcomes (such as local remissions, local disease-free survival, and overall survival) [74].

**Figure 3:**
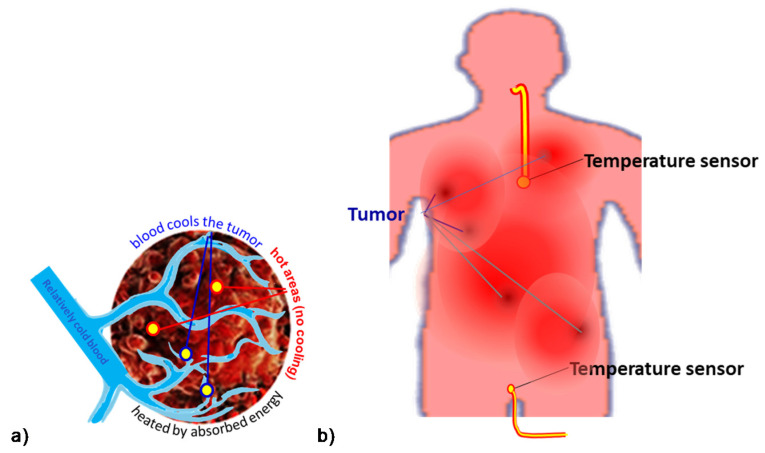
Challenges of temperature measurements: (**a**) the invasively inserted point sensors detect the very local temperature and not the average isothermal; (**b**) the semi-invasive temperature sensing catheters in lumens measure the temperature in near lumens, which could be far from the actual tumor temperature.

Measuring the isothermal situation, determining the $CEM\;43T_{x}$ dose has practical challenges. Reliable temperature measurement is an unachievable goal; Figure 3.

1. The invasive temperature sensors available are point detectors. When the point is near the arteries of a highly vascularized area, the temperature is less than in the low vascularization part, so many independent sensors are necessary to attain objective results. However, this induces safety and treatment problems.
2. Usually, a near lumen (such as the esophagus, bronchus, colon, or vagina) offers the possibility to approximate the temperature in the distant tumor, but this is again far from the reality in the target.
3. The most effective temperature mapping can be done with MRI measurement, using a phantom for reference, usually unionized water. The MRI measurement depends on the temperature, but also strongly depends on the structure of the measured volume. In the temperature measurement, both factors are included in calculating the result, but the calibration does not consider a final element: the changes in the structure, which is the goal of the LRHT treatment.

### 1.4. Challenge of the Heated Body

It looks evident that WBH offers the best heating possibility because of its easy control (measurements in body lumens) and the realized complete isothermal load on all the malignant cells and tissues. Notably, the WBH method does not show such good results in the high-temperature regime (≥41 °C). The prospective double-arm study shows that the overall survival was less in a combined hyperthermia application than in cases when only chemotherapy (ChT) was administered [72]. The same result was obtained in malignant pleural mesothelioma [73] when the toxicity was also higher in the combined treatments. Contrary to the 10+ times higher CEM 43 ℃ dose of WBH producing isothermal temperature ($CEM\;43\;℃\;T_{100}$), a fourfold development of metastases was measured in canine sarcomas with radiation therapy with or without WBH compared to the local heating [71]. The mild temperature WBH (mWBH<40 ℃ and $dose_{mWBH}$ < $\frac{2\;CEM43{}^{\circ}CT_{100}}{treatment}$) was effective [75]. (The additional parameter $T_{100}$ to CEM 43 °C denotes that 100% of the tumor received the dose). The mWBH activates the immune reactions, and so it could be a good complementary treatment for other therapies [76,77,78]. However, the demand for higher temperatures for direct cellular degradation challenges such applications and favors the LRHT application. Contrary to WBH, LRHT does not load the patient’s heart, and negligible electrolyte loss happens, and consequently, the inclusion criteria allow more patients.

### 1.5. Challenge of Homogeneity

The challenge of LRHT differs from that of WBH. While WBH ensured a homogeneous loading of the tumor, achieving homogeneity in LRHT is complicated. The well-focused heated volume spreads by heat-conduction over time, heating larger and larger body regions. The spread of heat triggers BF and so supports the delivery of necessary nutrients (glucose and others) to the tumor. A further challenge is an increasing difference between the BF of the tumor and its healthy host, BF to the host increasing much more quickly than in the tumor. This flow gradient promotes the invasion and dissemination of the cancer cells from the most vivid near-surface region of the proliferating tumor. An early phase III clinical study faced this problem, the straightforward local advances of HT + RT compared to RT alone not appearing in the survival time in breast tumors [79]. Another study obtained the same controversy: local remission success and the opposite in the overall survival [80]. The development of distant metastases was also observed [81]. The same reason led to a debate about LRHT results for the cervix, showing both advantages [17] and disadvantages [82] in survival.

A further study of cervix carcinomas supports the survival benefit [83], but again a critic has questioned this result [84,85]. Another phase III trial of cervical carcinomas with HT plus brachytherapy involving 224 patients noticed the same controversies between survival time and local control [86]. The controversy was observed in a study of locally advanced non-small-cell lung cancer (NSCLC) having a significant response rate improvement, although there was no change in overall survival [87]. A multicenter phase III trial for NSCLC also showed no improvements in overall survival in the hyperthermia cohort [88]. The cause was directly shown: the appearance of distant metastases was five times higher (10/2; *p* = 0.07) in the HT + RT group than in the RT cohort [88]. The study of the surface tumors had the same contradiction between the local control and survival rate [89].

Most likely, the improved dissemination of malignant cells forming micro- and macro-metastases causes contradictory results. We must learn from the contradictions and follow the admonishment of Dr. Storm, a recognized specialist of hyperthermia: “The mistakes made by the hyperthermia community may serve as lessons, not to be repeated by investigators in other novel fields of cancer treatment” [90].

Our task is to improve the controllability of LRHT, ensure the stable, successful applicability of heat therapy combined with RT in oncology, and fulfill the authentic promise that LRHT is an excellent complementary tool for RT [91]. Serious analysis is necessary as has recently been started [92]. I would like to continue this approach and add biophysical aspects. The data showing a highly significant improvement of local control obtained with LRHT and RT represent facts that we must consider as the basis for the further development of oncological hyperthermia and the correction of the problems with overall survival. We must concentrate on blocking invasion and reducing dissemination to overcome the issues. The task is to prevent the formation of metastases caused by heating. Furthermore, we may eliminate the metastases formed earlier, prior to thermal treatment, with the primary tumor’s local hyperthermia.

## 2. Materials and Methods

The radiation similarity of LRHT and RT induces the proposal to characterize the target volume with the isodose load. The isodose concept ensures reproducibility, safety, and efficacy too. The isodose in RT is simply the energy-dose of ionizing radiation measured in Gy ($=\frac{J}{kg}$) and applied to the tumor volume in daily fractions. The energy dosage may be reached in a session during a short time. The heating conditions limit the provision of the necessary energy. The LRHT needs a significantly longer time for a session than RT needs. Consider power, the applied energy per unit time (power, P [$\;\frac{J}{s}=W$]). The energy dose is the sum of the power $P_{i}$ during the time $\tau_{i}$ when it is applied ($E=\overset{t}{\underset{i=0}{\sum }}P_{i}\tau_{i}$). The power in the unit of mass is the specific absorption rate (SAR=P/m, where m is the mass of the target) measured in $\frac{W}{kg}$ units. The energy (E/m) is the dose considering the duration of the SAR load in the target, measured in $\frac{J}{kg}$ units, like the dose Gy in RT. In this way, the SAR offers the possibility to unite the doses of LRHT and RT. The energy increases the temperature, so in an ideal case, the SAR could be applied as the isothermal dose of LRHT.

The heating process starts with an approximately linear rate of temperature growth. It is quasi adiabatic. The relatively slow homeostatic feedback does not disturb the heating [93], and the SAR is proportional with this development in time (t): $SAR≅c\frac{dT}{dt}$ [31].

Physiological regulation and safety issues challenge this concept. The homeostatic regulation increases the BF in the targeted volume, and like a heat exchanger, cools it down. In this way, higher power is necessary than it otherwise would be desired without this physiological control. The systemic control increases rapidly and non-linearly [31] with different speeds as the BF changes. The treatment’s safety requires an intensive cooling of the body surface where the heating power penetrates. The cooling takes away a large amount of the applied energy, not contributing to the heating. The cooling and other energy losses (like radiation, heat diffusion, convection, etc.) limit the application of E as the dose because the actual energy absorbed in the body is uncontrolled. Consequently, temperature measurement is mandatory to estimate the amount of the absorbed power (SAR) in the target.

A new paradigm solves the challenge when the heating does not target the whole mass of the tumor, but the individual malignant cells are in focus [94]. This case avoids overly intensive feedback of the homeostatic regulation, and the various other losses also become more easily manageable. The individual cellular heating breaks the homogeneous isothermal requirement. The absorption is heterogeneous and microscopically individual, using the tumor’s natural thermal, electromagnetic, mechanical, and physiological heterogeneity [95].

The heterogeneous molecular actions in the selected volume do not contradict the isodose concept. The apparent contradiction originates from the false expectations of the isodose effect. The isodose does not mean that the action in the target involves all molecules and structures. It means that the isodose grants the desired molecular and structural changes in all isodose volumes. Nevertheless, the required molecular actions are individual and heterogenic. This homogenous-heterogenic vision is well observable in medication. When the body takes a dose intravenously, orally, or in other ways homogeneously in the body, the dose is calculated from the body’s volumetric parameter (BMI). However, the expected action of the drug is heterogenic, selectively targeting molecular structures. The ionizing radiation-activated DNA damage is the heterogenic goal of RT. LRHT targets other molecular effects, but the expected effect is incidental due to the averaging of the energy by the isothermal conditions.

**Figure 4:**
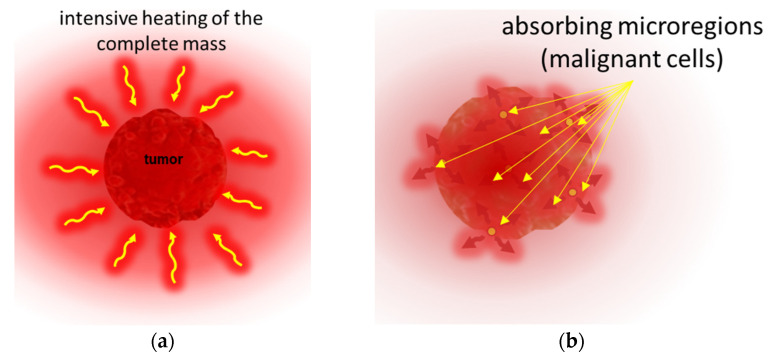
Draft presentation of the heating paradigms: (**a**) Homogeneous mass heating trying to achieve isothermal conditions. It intensively heats the surrounding healthy tissues as well. (**b**) Selective, heterogeneous (heterothermal) heating. It creates a high temperature in the absorbing points, but mild average temperature (<40 °C) in the surrounding healthy tissue.

The crucial point of the new paradigm is to select the malignant cells and concentrate the energy absorption upon them. The new paradigm is electromagnetic heating, as most applied hyperthermia methods use radiofrequency (RF) current. The current delivers energy to depth, its parameters (amplitude, frequency, and phase) being chosen optimally to find the heterogeneities produced by the malignant cells; Figure 4. All three parameters have dynamic changes by time variation, improving the selection mechanisms. The carrier frequency is amplitude modulated, and the modulation frequency is not constant, but follows the demands of the homeostatic control, representing a spectrum suitable for the spatiotemporal distribution of the cancer cells.

The heterogeneous heating has a crucial behavior: it provides a high temperature for the selected malignant cells, but the average temperature of the tumor remains under 40 °C. A temperature of over 40 °C downregulates the cytotoxicity of innate immune attacks [96,97], including those of the natural killer cells (NKs) [98]. On the other hand, substantial cellular thermal damage has been observed at temperatures above 41–42 °C [99]. Modulated electro-hyperthermia’s (mEHT’s) heterogenic heating could harmonize these two otherwise contradictory demands.

**Figure 5:**
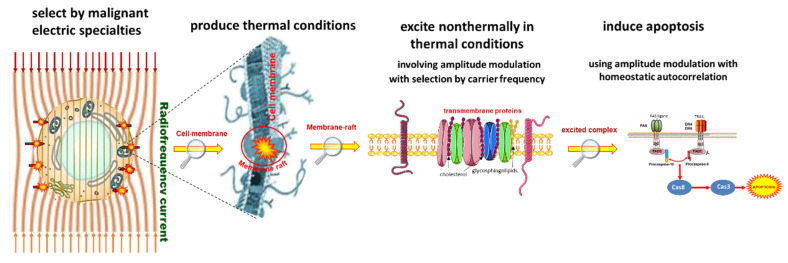
The transmembrane proteins of malignant cells absorb the energy in thermal and nonthermal forms. The amplitude-modulated carrier frequency’s nonthermal effect gives the apoptotic signal pathway (see below in results). The carrier frequency delivers the modulated signal and selects the malignant cells, while the modulation with homeostatic autocorrelation (time-fractal) constrains the apoptotic pathway.

Time-fractal modulated electro-hyperthermia (mEHT) supports the selection and induces programmed cell-killing processes, genuinely breaking the isothermal approach. Instead of homogenous heating of the target, mEHT uses excellent selection to force energy absorption on the malignant cells, heating them locally to the hyperthermia temperature to induce cellular changes in the targeted cells by thermal and nonthermal mechanisms [100]; Figure 5. The thermal component of the absorption heats the selected membrane rafts, which is the source of the temperature of the tumor, as is standard in heterogenic seeds or nanoparticle heating processes. In contrast, the nonthermal component causes molecular excitation for programmed cell death [101]. The excitation by electric field E has similar increase like the temperature increases the molecular reaction rate [102]. The cell-membrane represents decreasing impedance with increasing frequency, so the field penetrates the cell with improved intensity. The membrane practically shortcuts and does not significantly influence the RF current flow over ~25 MHz [103].

Nevertheless, the difference between the energy absorption between the membrane and intra- and extracellular electrolytes remains on high frequencies [104]. The primary energy absorption happens in the transmembrane proteins and their clusters on the rafts [105]. The density of membrane rafts is significantly higher than in the nonmalignant cells [106]. The absorbed energy makes the molecular excitation nonthermal and the temperature an essential joint conditional factor, promoting the reaction rate [107].

**Figure 6:**
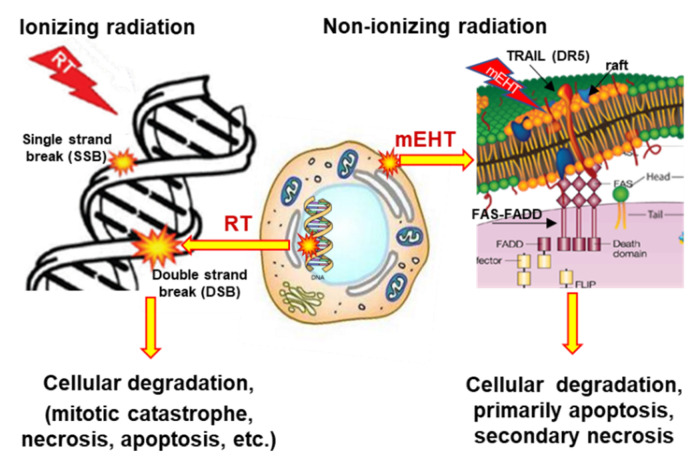
The conceptual similarity of RT and mEHT. Both therapies target molecular bonds, so the primary energy absorption is heterogenic. The result is cellular degradation in various ways.

The applied selective energy-absorption works like RT and realizes isodose conditions, too, concentrating on very local (nanoscopic) molecular effects, mostly to break the DNA strands in the isodose-defined volume. In this meaning, mEHT and RT have a similar nano targeting philosophy; Figure 6. The target is the natural heterogeneity of the tissues, as RT targets the DNA. The method recognizes the particularities of tumor cells’ microenvironment (TME) [108].

Two essential effects are considered for selection: thermal absorption and nonthermal excitation. The thermal component provides the appropriate temperature of the TME by heating the membrane rafts [105]. Another general thermal action affects the extracellular matrix (ECM) and a part thereof, the TME. This acts mechanically and molecularly [109], accompanying the thermal absorption of transmembrane protein clusters.

The nonthermal effect happens when “under the influence of a field, the system changes its properties in a way that cannot be achieved by heating” [110]. The nonthermal component excites the membrane receptors of the cells. The well-chosen electric current can deliver energy for molecular excitations involving various ionic and molecular interactions [31]. The process only has a subtle thermal effect and excites the molecules or structures that fit the applied resonant conditions [111].

The apoptotic signal by the mEHT excited membrane receptors and the apoptosis by the single or double-strand breaking of DNA for cellular degradation are strong similarities of RT and mEHT. Nevertheless, despite conceptional similarities, RT and mEHT have an essential difference: the additional thermal component in HT, which is absent in RT. Thermal absorption is mostly an unwanted side effect in ionizing radiation. The goal is only the molecular effects.

**Figure 7:**
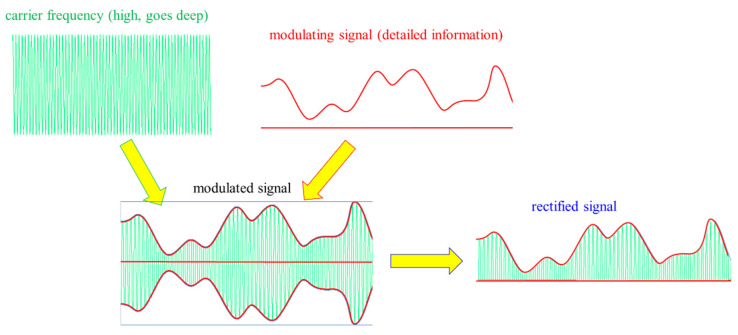
The modulation process compromises between the contradictory high- and low-frequency demands. The unification of the low-frequency modulating signal and the high-frequency carrier forms the modulated signal, a frequency spectrum on the carrier 13.56 MHz. The cell membrane rectifies and works for the excitation of apoptotic pathways. The high-frequency carrier gives the optimal thermal condition for the excitation by the low-frequency info signal in the selected cells.

The excitations of transmembrane proteins need low frequency [111], but their neuronal excitation, which may rise to 10 kHz [112], is not safe with the applied power. On the other hand, the frequency for selective heating is in the high RF frequency range. The mEHT solves the challenge of the contradictory simultaneous requirement of high and low frequencies. It uses the appropriate low frequency to modulate the high-frequency carrier; Figure 7 [113]. The membrane rectifies. The carrier frequency in the rectified signal remains active, but mainly at the cellular membrane (β-dispersion, see later). In this way, the original modulation signal makes the excitation process.

**Figure 8:**
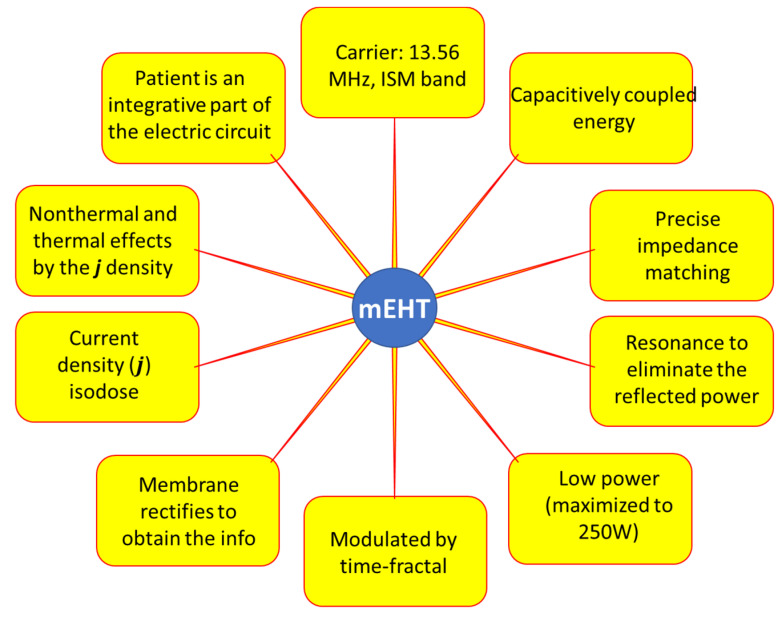
The technical conditions of mEHT. The realization of the method rigorously accommodates and utilizes the complexity of the heterogenic impact of mEHT to arrest the proliferation of cancer and degrade the developed tumor cells.

mEHT is a complex method, which complicates its technical realization. The technical details (Figure 8) need further explanation. I will discuss it in the discussion section of this article.

1. The chosen optimal carrier frequency is 13.56 MHz, which belongs to the freely applicable ISM band [114] and does not need shielding.
2. The energy is capacitively coupled, but it does not use the plane-wave approach. Plane-wave radiation is devoted to isothermal heating.
3. There is precise impedance matching [108] in the mEHT method. Proper impedance matching produces negligible reflected power (order of 1 W), mimicking the galvanic contact with the skin as much as possible.
4. It has resonant matching with micro-selection ability, which fits the impedance [109]. It eliminates the imaginary part of the impedance. It differs from the usually applied plane-wave matching.
5. The maximum adequate output power of mEHT is limited. The power limit depends on the size of the electrode. In device EHY2000+, the maximal power is 150 W, while in the model of EHY2030, which has optionally larger electrodes too, the limit is 250 W. The applied power in therapy depends on the localization and size of the tumor. The power limitation keeps the SAR less than for isothermal heating, but high enough to select and excite the membrane rafts of the malignant cells [100] and sensitizes to the RT [115,116].
6. The modulation spectrum is a low-frequency time-fractal [113], described by fractal physiology [117,118,119,120], which agrees with the homeostatic molecular temporal balance [113]. mEHT extensively uses the modulation technique to identify fractal structures in space and time (dynamics) in spatiotemporal identification [113]. The electric parameters (resistance and capacity) depend on the malignant status [121]. The selection between malignant and healthy cells was measured as a characteristic time-fractal [122]. The modulation delivers temporal information executing enzymatic processes at the cell membranes [123], promoting the consequence of the excitation.
7. The membrane rectifies [124,125], and considerably gains the strength of signal intracellularly [103,104]. The rectified signal acts in the low- and high-frequency ranges.
8. The correct impedance matching provides an appropriate electric field that ensures the current density (j). The j is the parameter of the isodose conditions, ensuring the constant current density in the target. A complex value describes the current depending on the phase shift from the applied signal voltage. The dominant dielectric actions (heating and excitation energies) produce thermal and nonthermal effects.
9. The modulated j-current density actively produces both the thermal and nonthermal effects.
10. The patient is interactively connected to the electric circuit, like a discrete element of the RF-net. This solution allows the real-time control of the patient due to the treated tumor being actively sensed and targeted as part of the tuned electric circuit.

Further technical details can be found elsewhere [108].

## 3. Results

The mEHT method is the focus of intensive research regarding all attributes. Phantom experiments show the proof of the thermal concept, measuring the temperature development in well-chosen chopped-meat phantoms [126,127], and computed results show the validity of heat selection using tissue heterogeneities, also proven in experimental setups [128].

These macro approaches are well completed with the micro-approach, calculating the nano-range thermal and nonthermal components [105].

In vitro experiments fixed the thermal effects to the reference calibration using the U937 human lymphoma cell line [95], and the HT29 and A431 [94] cell lines. The quantitative dose equivalence of mEHT with RT defines the harmonizing basis of cellular degradation in two different lung cancer cell lines, A549 and NCI-H1299 [129].

mEHT is a mild LRHT in the conventional meaning. The temperature dynamically grows in the mass of the liver when there is no tumor inside because selective targeting does not modify the distribution, as temperature measurement in the liver of an anesthetized pig shows [130]. The thermal component of mEHT heats the target, which may be used for temperature mapping in a preclinical murine model [131] at a mild level. A mild hyperthermia temperature level in humans could be measured in cervical cancer, which increases the peritumoral temperature to 38.5 °C, with proper blood flow for the complementary treatments [132].

The comparison of mEHT to wHT and to plane-wave fitted, non-modulated capacitive hyperthermia (cHT) at the same temperature shows a significant improvement of apoptosis with mEHT in the HepG2 cell line [133]. It showed that the wHT and cHT (the homogeneous heating) cause approximately the same low apoptotic rate, which reveals the advantage of the mEHT heterogeneous concept. The breaking of DNA measured with subG1 also significantly improves with mEHT as compared to the conventional homogeneous methods [133]. Radioresistant pancreatic cell lines show extensive DNA fragmentation measured with subG1 after mEHT [134].

**Figure 9:**
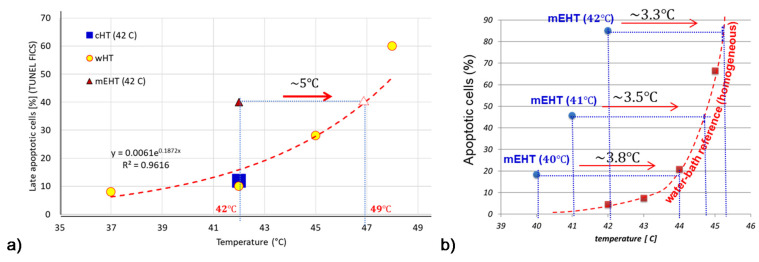
The calibration of the thermal factor of mEHT. (**a**) The homogeneous HT (water-bath hyperthermia, wHT) is used to calibrate apoptosis. The mEHT causes effective apoptosis at 42 °C, corresponding to the calibration at 5 °C higher (HepG2 cell-line) [133]. The mEHT affects the rafts on the cell-membrane with a 5 °C higher temperature than the average medium indicates. (**b**) Another calibration measurement with the U937 cell line [95,136]. The mEHT shows a >3 °C temperature difference in apoptotic efficacy at all measured points.

The effect has given a possibility to make a reference calibration of mEHT compared to wHT on HepG2 cells shown at ~5 °C [133], while in the U937 cell-line [95], it shows a >3 °C shift to the advantage of mEHT over wHT (Figure 9), it is supposed that the difference indicates a 3+°C higher temperature of rafts than of the TME. The gain of tumor destruction at 42 °C is ≈4.9 fold, which corresponds well with the in vivo experiments (≈4.3) in HT29 colorectal carcinoma [135].

A critical thermal factor is that the possible touching point of two cells has a drastically increased heat-production due to the extensive SAR at that point [105]. The telophase of the cell cycle naturally forms a tight touching of the two just-created daughter-cells, where the increased SAR could block the finalizing of the cycle and cause the daughter to degrade [137]. Like all complex phenomena, the cytoskeleton’s effect could also act oppositely. The reorganization of actin filaments and microtubules by an outside modulated electric field can support the proper polymerization of the cytoskeleton when the cell is only pre-malignant [138]. The close independent malignant cells attract each other by the induced dielectrophoretic forces and the vast electric field gradient between the cells [105]. This makes it possible to reconstruct the intercellular E-cadherin connection, allowing the regular networking of the cells [133]. The deformation of the cells by external field depends on the frequency [139]. The carrier of mEHT is high enough that the deformation is negligible due to the higher conductivity in the ECM than in the cytoplasm [104].

The molecular models concentrate on the membrane effects, showing the thermal and nonthermal results. The same heat conditions force the same processes in the cytosol ER and other cellular organelles, and the heat-sensitive transient receptor potential vanilloid receptor (TRPV) also senses the same temperature for action. The excess ionic concentration is caused by mEHT [140], which increases the influx of ${\mathrm{Ca}}^{2+}$ ions from the ECM to the cytosol. The high $i{Ca}^{2+}$ promotes apoptosis in the mitochondria-dependent intrinsic signal pathway [141]. The decreased membrane potential of mitochondria [136] well supports the mitochondria-associated apoptotic process. The mEHT induces the ${\mathrm{Ca}}^{2+}$ influx with the assistance of E2F1 [142], which regulates the HSPs without heat-shock [143], supporting the possible factors of the nonthermal effect of applied electric current.

Research of the nonthermal effects on HT29 and SW480 human colorectal cancer cell lines shows a significant nonthermal impact on the ionic fluxes, and mEHT has doubled the antiproliferative and anticlonogenic effects of conventional water-bath heating (wHT) at 42 °C [144].

There are tumor-specific thermal and nonthermal stresses with mEHT related to the metabolic profiles of the targeted malignant cells having elevated glycolysis [145]. The efficacy of mEHT may correlate with the tumor metabolic profile by the targeted selection [146].

The nonthermal activity causes structural changes affecting the intracellular polymerization of filaments [138]. The fluctuations also have an essential role in the electromagnetic interaction, showing thermal and electric noise limitation in the TME connected membrane [147].

mEHT applications focus on induced apoptosis [148,149]. The method may cause caspase-dependent paths through Cas8 (extrinsic way) and Cas9 (mitochondrial, intrinsic way) [133,150] and independent [151,152] apoptosis. A notable factor is the arrest of the XIAP effect to block the main path of caspase-dependent apoptosis by the secretion of SMAC/Diabolo [153] and Septin4 [154].

Experiments show that the aggressively radioresistant cell (L9) could be resensitized by mEHT [155], and also, radio-resistive pancreatic cells (Panc1, Capan1) show extended apoptosis when treated with mEHT [134,156,157]. mEHT also destroys these adenoma-carcinoma cell lines [148]. The radiosensitization of mEHT significantly intensifies the autophagy and apoptosis in SCC VII and SAS cell lines compared to RT and wHT [158]. The massive apoptotic activity could be used for thermal dose calibration and energy-absorption-based temperature mapping [159].

Curiously, a notable reduction of apoptosis was measured with the addition of artificial gold suspension nanoparticles (NPS) to the targeted volume [160].

DNA fragmentation drives tumor-cell degradation [152]. The induced stress by mEHT upregulates the tumor suppressor p53 protein, a cell-cycle regulator, one of the key cell-cycle regulation and DNA repair players. mEHT activates DSB production. The phosphorylated form of histone family member X (γH2AX) as a DSB marker can activate p53.

mEHT significantly upregulates the γH2AX producing DSB in treating a B16F10 melanoma murine tumor model [161], in C26 colorectal allografts [101]. The subG1 cell fraction grows significantly in a radioresistant ductal adenocarcinoma cell-line (Panc1) combined with mEHT + RT 24 h posttreatment [134]. In the same study, the cellular viability drastically decreased in these resistant tumors in mono and complementary therapies with mEHT. As independently expected [162], the thermal component of mEHT acts in synergy with the electric excitation, affecting the repair of DNA. The induced upregulation of cyclin-dependent kinase inhibitor protein ($p21_{waf1}$) and the reduced Ki67 proliferation marker correlates with γH2AX, showing that the DSB is related to mEHT treatment [101,163]. The suppression of Ki67 and the significant growth inhibition has been shown in breast cancer murine isograft [164].

The heatmap of the gene expression chip shows the gene regulations of the mEHT-treated samples in an HT29 xenograft [165], in various gliomas [142], and also in vitro in the U937 cell line [136]. The gene map shows a distinct difference in the gene regulations between the homogeneous wHT and inhomogeneous mEHT treatments [136] at the same 42 °C temperature.

Extended research deals with the possible tumor-specific immune processes of the heterogenic thermal and nonthermal effects and supports the emerging science of immuno-oncology. This examination’s direction is focused on the abscopal effect, an emerging approach in RT research [166], also recognized by the ASCO [167]. The expectation is a tumor-specific immune situation, considering that cancer precludes regular immune attacks. The mEHT being concentrated on the tumor cells provides immunogenic information for the adaptive immune system about the malignant state and simultaneously sensitizes the tumor to the innate immune attack. This situation could extend the RT + mEHT local synergy to be active in the entire system.

The research concentrates on the optimal liberation of the genetic information from the cancer cells during their degradation. We found that the best process to achieve our goals is “soft” killing, not degrading the secreted molecules with too large an energy load. So, we suppressed the necrosis and the observed apoptosis based on the immunogenic efforts. One particular type of apoptosis, immunogenic cell death (ICD), was the aim, which is associated with a damage-associated molecular pattern (DAMP) as expected in the abscopal activity of RT too [168]. The promotion of damage-associated molecular pattern signals in an HT29 xenograft clearly showed a DAMP when treated with mEHT [165]. In parallel research, the innate NK*-cell activation to attack the selected malignant cells was also proven in A2058 melanoma in a murine xenograft model [169].

DAMP productive mEHT has been supported with various immune supports, which otherwise had no impact on cancer alone. The support by dendritic cells (DCs) has shown to be an excellent addition to mEHT, despite its inactivity alone. The combined treatment showed a perfect abscopal effect on the preclinical murine model, using SCC VII malignant cell inoculation to the animal [170], detecting $CD3^{+}$, $CD4^{+}$, and $CD8^{+}$ T-cells resulting from DC maturation create antigen-presenting cells (APCs), increasing the S100 DC marker [171]. The presence of killer-T-cells ($CD8^{+}$) increased significantly. The mice had two distant tumor lesions (in the femoral and chest region) modeling metastases. The femoral region was treated, and the chest remained untreated. After multiple treatments, an apparent abscopal effect was observed, and the tumor growth was completely blocked in the untreated chest tumor and the treated femoral [170]. Importantly the $T_{reg}$ protumoral activity was blocked as well, measured with Foxp3 suppression.

The abscopal effect of multiple mEHT treatments alone has been shown in B16F10 melanoma pulmonary metastases, where a significant anti-tumor effect, reducing the number of pulmonary metastatic nodules, and high immune cell infiltration was also present [163].

Similar results were obtained in another study, significantly improving the immunological tumor microenvironment with mEHT followed by dendritic cell immunotherapy [172]. This study also showed that no immune-effect happens with wHT at the same 42 °C temperature. A remarkable result of this study was that the rechallenge of the cured animals with the same malignant cell-line was rejected, observing the adaptation of the immune system, behaving like “tumor-vaccination”.

A natural herbal immune-support, Marsdenia tenacissima (MTE), caused a similar arrest of the tumor development systemically after mEHT, despite it being ineffective alone [173,174].

mEHT’s combination with the simple conventional tumor-suppressive drug Doxorubicin (Dox) shows a robust immune activation observed with ICD, DAMP, and APC production and having a solid synergy with mEHT in intensively producing DSB, measured by γH2AX [175].

The starting point of human applications is safety. One of the most sensitive organs, the brain, was tested by dose escalation to measure the safety in human glioma cases, proving the safety of mEHT [176]. Many RT-related clinical therapies combine the heat effects with radio-chemotherapy (ChRT). The reason is to be effective systemically by using the drug when LRHT and RT are only local. The ChRT could be a complete game-changer because the reaction rate of chemo-agents exponentially rises by reciprocal temperature (Arrhenius law) and makes cell death independent from RT or HT effects.

A Phase III trial comparing randomized cohorts of ChRT ± mEHT in clinical practice showed an excellent response to the combination with mEHT compared to the ChRT alone [177], and the toxicity was also low [178]. The abscopal effect was directly measured in addition to the Phase III study [179,180], showing a significant increase compared to the otherwise expected systemic effect of the ChRT. RT in combination with mEHT with checkpoint inhibitors also shows the abscopal effect in various tumors [116], supposing the immune-modulator function of mEHT [181]. Tumor-directed immunotherapy in the combination of RT and mEHT is also a possible option [182]. Table 2 lists 25 studies using mEHT complementarily to RT or ChRT, but the complete study list also contains monotherapy and chemotherapy.

Some recent reviews are available for references regarding conceptual [31,111], technical [94,108], preclinical [101,108], and clinical [183,184,185] aspects of the mEHT method, showing its efficacy in oncology.

**Table 2:** The table refers only to the clinical results obtained with mEHT complementary to RT or ChRT.

No.	Tumor Site	Number of Patients	Treatment Used	Results	Reference
1	Advanced gliomas	12	mEHT + RT + ChT	CR = 1, PR = 2, RR = 25%. Median duration of response = 10 m. Median survival = 9 m, 25% survival rate at 1 year.	Fiorentini, et al., 2006 [186]
2	Various brain-gliomas	140	mEHT + RT + ChT	OS = 20.4 m. mEHT was safe and well tolerated.	Sahinbas, et al., 2007 [187]
3	High-grade gliomas	179	mEHT + RT + ChT	Longstanding complete and partial remissions after recurrence in both groups.	Hager, et al., 2008 [188]
4	Glioblastoma & Astrocytoma	149	mEHT + RT + ChT (BSC, palliative range)	5y-OS = 83% (AST) in mEHT vs. 5y-OS = 25% by BSC. 5y-OS = 3.5% in mEHT vs. 5y-OS = 1.2% by BSC for GBM. Median OS = 14 m of mEHT for GBM and OS = 16.5 m for AST.	Fiorentini, et al., 2019b [189]
5	Advanced cervical cancer	236	Random. Phase III (RT + ChT ± mEHT [preliminary data]	Preliminary data for the first 100 participants. A positive trend in survival and local disease control by mEHT. There were no significant differences in acute adverse events or quality of life between the groups.	Minnaar, et al., 2016 [190]
6	Advanced cervical cancer	72	mEHT + RT + ChT	CR + PR = 73.5%; SD = 14.7%. The addition of mEHT increased the QoL and OS.	Pesti, et al., 2013 [191]
7	Advanced cervical carcinoma	20	mEHT + RT + ChT	mEHT increases the peri-tumor temperature and blood flow in human cervical tumors, promoting the radiotherapy + chemotherapy	Lee, et al., 2018 [132]
8	Advanced cervical carcinoma	206	Random. Phase III (RT + ChT ± mEHT) [abscopal effect]	The abscopal effect grows significantly with mEHT complementary to ChRT.	Minnaar, et al., 2020 [178]
9	Advanced cervical carcinoma	206	Random. Phase III (RT + ChT ± mEHT) [toxicity & Quality of life]	mEHT does not increase the toxicity of ChRT but increases the quality of life	Minnaar, et al., 2020 [178]
10	Advanced cervical carinoma	202	mEHT + RT + ChT	Six-month local disease-free survival (LDFS) = 38.6% for mEHT and LDFS = 19.8% without mEHT (*p =* 0.003). Local disease control (LDC) = 45.5% with mEHT LDC = 24.1% without mEHT; (*p =* 0.003).	Minnaar, et al., 2019 [177]
11	Advanced NSCLC	97	mEHT + RT + ChT	Median OS = 9.4 m with mEHT OS = 5.6 m without mEHT; (*p* < 0.0001). Median PFS = 3 m for mEHT and PFS = 1.85 m without mEHT; *p* < 0.0001.	Ou, et al., 2020 [192]
12	Advanced NSCLC	311 (61 +197 +53)	mEHT + RT + ChT	Two centers PFY (n = 61), HTT (n = 197) control (n = 53). 80% (PFY), 80% (HTT) had distant metastases, conventional therapies failed. Median OS = 16.4 m (PFY), 15.6 m (HTT), 14 m (control); 1st y survival 67.2% (PFY), 64% (HTT), 26.5% (control).	Dani, et al., 2011 + Szasz, 2014 [193]
13	Advanced rectal cancer	76	mEHT + RT + ChT	Downstaging + tumor regression, ypT0, and ypN0 were better with mEHT than without. No statistical significance.	You et al., 2020 [194]
14	Various types of sarcoma	13	mEHT + RT + ChT	Primary, recurrent, and metastatic sarcomas responded to mEHT, the masses regressed.	Jeung, et al., 2015 [195]
15	Advanced pancreas carcinoma	106	mEHT + RT + ChT	After 3 m, PR = 22 (64.7%), SD = 10 (29.4%), PD = 2 (8.3%) with mEHT after 3 m of the therapy. In group without mEHT in the same time: PR = 3 (8.3%), SD = 10 (27.8%), PD = 23 (34.3%). The median OS = 18 m with mEHT and OS = 10.9 m without mEHT.	Fiorentini, et al., 2019 [196]
16	Advanced pancreas carcinoma	133 (26 +73 +34)	mEHT + RT + ChT	Two centers PFY (n = 26), HTT (n = 73) control (n = 34). 59% (PFY), 88% (HTT) had distant metastases, conventional therapies failed. Median OS = 12.0 m (PFY), 12.7 m (HTT), 6.5 m (control); 1st y survival 46.2% (PFY), 52.1% (HTT), 26.5% (control) QoL was improved.	Dani, et al., 2008 [197]
17	Metastatic cancers (colorectal, ovarian, breast)	23	mEHT + RT + ChT	OS and time to progression (TTP) were influenced by the number of chemotherapy cycles (*p* < 0.001) and mEHT sessions (*p* < 0.001). Bevacizumab-based chemotherapy with mEHT has a favorable tumor response, is feasible, and well-tolerated for metastatic cancer patients.	Ranieri, et al., 2017 [198]
18	Rectal cancer	120	mEHT + RT + surgery	In mEHT group, 80.7% showed down-staging compared with 67.2% in non-mEHT group.	Kim et al., 2021 [199]
19	Gliomas	164	mEHT + RT + ChT	CR + PR is 41.4% for mEHT and 33.4% for conventional therapies.	Fiorentini et al., 2020 [200]
20	Ovarian, cervical cancer		mEHT + RT + ChT	The feasibility and success of oncothermia is proven.	Wookyeom, et al., 2018 [201],
21	Various sites	784	mEHT + RT + ChT + surgery	Preliminary results show promising survival trajectories. mEHT is a safe treatment with very few adverse events or side effects, allowing patients to maintain a higher quality of life.	Parmar et al., 2020 [184]
22	Various sites		mEHT + RT + ChT	Planned trial.	Arrojo et al., 2020 [202]
23	Various sites		mEHT + RT + ChT	The feasibility and success of oncothermia are proven.	Szasz AM et al., 2019 [183]
24	Advanced glioblastoma	60	mEHT + RT + ChT	No added toxicity by immunotherapy. Median progression-free survival (PFS) = 13 m. Median follow-up 17 m, median OS was not reached. The estimated OS at 30 m was 58%.	Van Gool, et al., 2018 [203]
25	Different types of metastatic/recurrent cancers	33	mEHT + RT	CR = 2 (6.1%), Very good PR = 5 (15.2%), PR = 13 (39.4%), SD = 9 (27.3%), PD = 4 (12.1%). Three patients (9.1%) developed autoimmune toxicities. All these three patients had long-lasting abscopal responses outside the irradiated area.	Chi, et al., 2020 [116]

## 4. Discussion

All complex therapies overcome a contradictory process by considering one of the robust behaviors of this complexity: self-organization and the consequent self-similarity [204]. Recent decades have seen the development of various approaches describing the complexity of systems with self-organization [205,206]. The homogenous approach does not consider the natural heterogeneity of complex living systems. mEHT applies the selection of microtargets to distinguish the various parts and functions of the living organism.

### 4.1. The Electromagnetic Selection

The selection at the macro scale uses the intensive metabolic activity of the malignant cells to produce increased ionic density in the TME of the cells. In this way, the entire tumor has a higher complex conductivity ($\sigma^{*}$) for the electric current than its healthy environment [105,207,208,209,210]. The conductivity is proportional with the imaginary part of the complex dielectric function ($\varepsilon^{*}$), depending on the ionic density (strength) of the target. A part of the high conductivity could be followed using positron emission tomography (PET). The PET measures the intensified glucose metabolism, producing enhanced ionic concentration (primarily lactic acid). The PET results could be considered in the planning of RT [211], as it is a good addition for mEHT seeing the tumor activity, which is connected to the selectivity of the method. The electric current will choose the most accessible route (the most conductive one), flowing through the tumor.

Another electromagnetic selection mechanism concentrates on microregions (TMEs) using distinct structural heterogeneity. The individual autonomic development of cancer cells weakens the intercellular connections, breaking the E-cadherin protein connections. The malignant processes’ breaking of the networking order also differentiates them in this parameter. In this way, the TME starts becoming gradually disordered by the development of the malignant network-breaking character shown in early observations by NMR measurements [212,213,214]. The disorder increases the dielectric permittivity (ε) of the microregion [215,216,217,218]. The high ε drives the mainly chosen radiofrequency (RF) current like the high σ does. The plasma membrane and the TME absorbs the central part of the energy in the MHz region of the RF current [104]. The microregion of the tumor cells has considerable gradients of the electrolyte constituents of the electrolyte. The TME is in direct contact with tumor cells, containing molecular bonds to the membrane surface, while ECM is wide. Its primary function is connected to the transport processes. The water content of the TME interacts with the membrane [219], having variant bonds [220], and critically alters the membrane effect, showing a low SAR but high voltage drop [221], which can help the signal’s excitation of the raft proteins [222]. The electrostatic charge of the membrane attracts the ions from the ECM, whose very different effect is sufficient to establish a transmembrane potential [223].

The rafts operate as a trigger of the cellular processes [224]. The rafts collect dynamic proteins [225], including proteins with high lateral mobility in the membrane [226]. The cataphoretic forces generated by modulated electric fields induce lateral movements and are sensed by the rafts in the membrane [140]. The size of these clusters is in the nano range. It depends on the ratio of protein to lipid content, different ranges of their horizontal diameters have been measured: 10–100 nm [227]; 25–700 nm [228]; 100–200 nm [229]. The width of the membrane is 5 nm [230], but the thickness of rafts, due to their transmembrane proteins, has a larger size. Note that the temperature increase of the nanoparticle (NP) is proportional to the square of its radius [231], which gives an easy comparison of the temperature using the sizes of the particles. The standard applied SAR in nanoparticles, considering their weight heating is 100–1500 MW/kg [4]. The mEHT heats not only the rafts but heats the TME and also the tissues to a lesser extent. Rough approximation of the absorbed power of rafts by mEHT is SAR > 1 MW/kg [105]. However, the role of absorption differs in nanoparticle and raft heating. The absorbed energy in nanoparticles produces only heat, while in the rafts with excitable structures, the energy divides into thermal and nonthermal effects.

The relatively large rafts contain approximately half of the membrane mass because of their relatively large mass compared to the lipids, representing only 2% of the membrane components [104]. The targeting of the rafts induces accurate energy absorption. The incorporation of energy happens at clusters of transmembrane proteins [95,140]. The temperature of the selected rafts is over the thermal averaging of the tissue. On average, the relatively small SAR is high in the rafts, similarly to the nanoparticle selective heating.

The selection of mEHT is demonstrated in an experiment with artificial NPs added from suspension to the targeted volume [160]. The injecting gold NPs or other artificial good energy absorbers produce a higher quantity of energy absorption in the target. The temperature grows by the diffuse heating from these too. Despite the more intensive energy absorption, the observed apoptosis in these cases decreases [160]. Probably, the sharing of the energy between the membrane rafts and the NPs causes this contradictory effect. The phenomenon supports the proofs of the selection by mEHT.

The selection appears in the ECM too. The current which flows in the extracellular electrolyte heats it more in the areas of selected TMEs than in the membrane-isolated cytosol. The energy analysis of the heating differences explains how this effect contributes to cell-killing mechanisms [109].

Well-defined conditions limit the SAR in the target, which limits the average power provided.

1. The thermal effect happens in nanoscopic local “points”, the rafts. These NPs are molecular clusters and sensitive to overheating. When the absorbed energy is too large, it destroys the rafts by overheating. The mEHT loses its most significant advantage, the excitation of signal-transports for apoptosis and immunogenic cell death (ICD).
2. The selection mechanisms of mEHT also limit the SAR, which forces temperature development. At high temperatures, the heat spreads extensively, and the microscopic differences vanish on average. A macroscopic average will characterize the target, as in WBH. The limited energy absorption is mandatory for the selection of rafts.
3. The appropriate frequency is selected around 10 MHz [94]. When the frequency is larger (>15 MHz), the membrane impedance becomes too small to select the disordered TME accurately. The current will flow through the entire target tissue almost homogeneously, neglecting the selection heterogenic selection factors of malignant cells. When the carrier frequency does not ensure selection, the modulation also activates the healthy cells. The significantly larger amount of membrane rafts between healthy and malignant cells [106] remain selective factors only.

### 4.2. Nonthermal Processes

Healthy dynamism realizes a certain and strictly ordered set of molecular signals in space and time to maintain homeostatic control. The functional signals repeatedly correlate with the given functions (for example, the metabolic cycles), causing an autocorrelation of the resultant signal [232,233]. Note that spatial autocorrelation is a valuable tool in studying the microarchitecture of TME [234]. A significant periodic component in a data set has data points in a time series that correlate with the preceding data points in time, consequently measuring the self-similarity of different delay times in the signal. The autocorrelation could be simply visualized in the particular self-overlapping value of the signal (how the signal correlates with its earlier values). Hence, when the signal is shifted with a time lag, it correlates with earlier values.

The autocorrelation makes preferences of bioeffect variants [235], changing chemical reactions, selecting them by their timing, and ordering them by the time required for the desired signal-pathway or enzymatic actions. The biological effects happen on a broad time-scale. An adequately chosen time-fractal modulation promotes the desired autocorrelation of the signal. This modulation noise regulates the biosystems to their normal homeostasis [236], and the spatial autocorrelation also ensures the harmlessness of white-noise excitation [237]. On the other hand, the otherwise healthy support has an opposite impact on malignant processes. It does not harmonize with the malignant processes, is absorbed in an anharmonic way (heating), and does not excite the molecular signals. The modulation signal selectively supports or blocks the cellular membrane’s preferred (healthy) or avoidable (malignant) processes. This dynamic effect expands the electrodynamic selection mechanisms, taking effect not only in structural but also in dynamical malignant irregularities in the health system. Both the structure and dynamics of living organisms have a fractal pattern. The spatiotemporal structure and its consequence, the signal character measured by the fluctuations, differentiate malignant tissue from healthy [121] and are measurable by the RF current [122]. The fluctuation difference between malignant and healthy tissues grounds the applied modulation on the RF carrier. The mEHT therapy uses a pattern recognizing and harmonizing fractal modulation [113] to keep the natural homeostatic control as effective as possible. The well-chosen fractal modulation favors the healthy homeostatic control and combats malignancies outside this regulation [113]. The applied modulation in mEHT considers the natural heterogeneity in space and dynamics, including the autocorrelation of living processes.

Depending on the RF frequency, various processes happen in biomaterials, described by frequency dispersions [238]. The α-dispersion covers the low-frequency interactions (~10 Hz–~10 kHz). This dispersion affects the molecules near the cell membrane interacting with the TME, the various membrane components, and the transmembrane proteins. Ionic electrodiffusion affects the dielectric loss of bound water in molecules. Intercellular charging appears as the main change in α-dispersion. This region signifies our excitation activity. However, its direct application is limited by its missing selectivity and the risk of dangerous nerve stimulation. The task was to find a frequency that selects, does not make nerve stimuli safe, and penetrates deeply into the body. The higher frequencies are satisfactory, and the combination of those with low frequency in modulation solves this complex problem by applying 13.56 MHz carrier frequency and modulating it with a spectrum of frequencies in α-dispersion range.

The 13.56 MHz belongs to the β-dispersion. The broad range of β frequency dispersion [111,239] (known as the interfacial polarization effect) allows selective treatment [240].

The chosen 13.56 MHz select the cellular formations [241] interacting with the interface of membrane-electrolyte structures, using Maxwell-Wagner relaxation [239] causing interfacial polarization of the cell membranes [242]. It changes the charge distribution at the cellular or interfacial boundaries [219]. A part of β-dispersion takes effect in the torque of biological macro-molecules (like proteins) and orients these contrary to the thermal background [243].

The range of the δ-dispersion [244,245] overlaps with β-dispersion interacts with the dipolar moments of proteins and other large molecules (like cellular organelles, biopolymers) [246], and affects the suspended particles in TME [247]. The δ-dispersion is primarily selective for water-bonded lipid-protein complexes in the membrane rafts [219].

Important practical point to choose the carrier frequency in the β/δ interval, and internationally approved for industrial, scientific, and medical use. A total of 13.56 MHz was ideal for these requests. The model calculation also shows the importance of the 13.56 MHz [248]. The electrolyte and membrane differences between the malignant and healthy tissue [249,250] are involved in the selection. The membrane lipid targeting has recently come into focus, and it is recognized as having potential for cancer therapy [251]. Note that the rearranging (disordering) of the water structure at the membrane is clearly visible in the absorption spectra and needs energy [252], which could be obtained from the RF current density.

The carrier frequency’s RF energy ensures the selection and absorbs on the membrane rafts [105]. The modulation in α-dispersion makes the requested excitation affects their receptors [140], which destructs the malignant cells dominantly in an apoptotic way [253]. Theoretical considerations also prove the nonthermal effect of mEHT, showing that the observed effects could not have a solely thermal origin [254]. The physical origin is also explained [255] and centers on the effect of the modulation.

The bioelectromagnetism determines various features of homeostasis [256]. The modulation is not a single frequency. It is a spectrum of 1/f spectral density in the audio range (<20 kHz), improving the electric field’s homeostatic connection by a similar time-fractal structure. The autocorrelation of the signal prefers the external apoptotic pathway. The membrane gains the rectified signal [106], so the 10% modulation depth satisfies the expected signal excitation. The adaptation of this spectrum is in its 1/f (“pink”) noise structure [236,237] which depends on the target and automatically modifies the effect of modulation by the noise structure in the TME [147].

This dynamic selection and distortion of malignant cells detect and treat. In this way, the mEHT is a kind of theranostic method.

### 4.3. Effect of RF Current Density and the Dynamic Heating

Impedance-matched mEHT uses the current density j as an isodose parameter. The current density does not depend on the technical losses outside of the target. It considers only the power which goes into the body. The isodose of j is approximative. It is rigorously true only for homogeneous targets. A large average statistically offers a quasi-homogeneity. This homogeneity expectation is a typical challenge in doses of chemotherapies, which expect the homogeneously transported drug in the body, which selectively destroys the malignancy. In the mEHT method, the same challenge appears in the homogeneity concept.

The j depends on the conductivity (σ) and the electric field strength vector (E): $j=\sigma E\;\left(\frac{A}{m^{2}}\right)$. The j vector and the σ conductivity are complex numbers, and due to the biomaterial not being a perfect conductor, it is lossy. The electric field drives both the thermal heating and the nonthermal excitation processes, and it is linearly proportional with the complex j, ($E=\frac{1}{\sigma }j$) so the current density well describes the amount of excitation, so linearly generates a nonthermal effect. In a good approximation, j does not depend on the size of the applied capacitor plates. The size of the plate defines the area $A=r^{2}\pi$ of the circular electrode with radius r. The current (I) depends on the electrode voltage (V) and the resistivity (R) between the electrodes: $I=\frac{V}{R}$. The current density $j=\frac{I}{A}$ while $R=\frac{d}{\sigma A}$, where d is the distance between the electrodes. Consequently, $j=\frac{V\sigma }{d}$, depends on only the constant parameters and does not depend on the area or radius of the electrode. The j can be kept constant when the electric potential is constant. The volume between the plates has an equal dose, as with the homogeneity principle of systemic chemotherapy.

The power (P) as the absorbed thermal energy depends on the square of the field: $P=\sigma E^{2}=\frac{1}{\sigma }\;j^{2}\left(\frac{W}{kg}\right)$. In homeostatic conditions, when the general energy loss is negligible, the measurement of the incident power (correlation with $j^{2}$) offers a dose identification. The dose, in this case, is the time summary of the power ($dose=\frac{energy}{mass}=\int^{\;}Pdt=\int^{\;}\frac{j^{2}}{\sigma }dt$, [$\frac{J}{kg}$]). The high efficacy of current matching [257], and the low value of the cooling energy-loss allows this simple dose monitoring [68,258] instead of by the local temperature. Consequently, mEHT has no compulsory demand to measure the temperature. It has enough accuracy to measure the absorbed energy by the incident, not forced RF current density [68].

**Figure 10:**
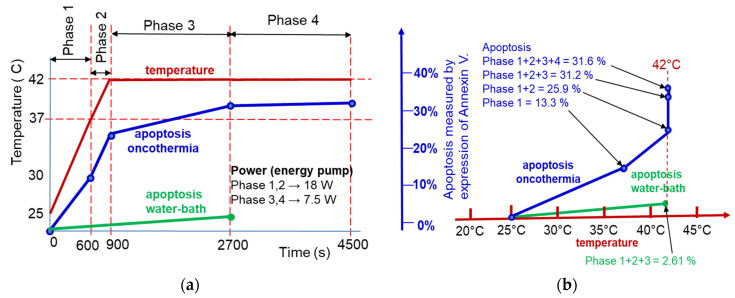
The effect of heating and maintaining the temperature on apoptosis. The mEHT had significantly higher apoptotic cells than the wHT at the same temperature. (**a**) The apoptosis saturated when the temperature became constant at the temperature maintenance period of treatment. (**b**) The temperature dependence of apoptosis shows a limit at the saturated temperature.

When the temperature grows, the heating period demands a higher dose than when keeping the temperature constant [150,159]. The higher power increases the dose by $j\sim \sqrt{P}$. The heating excites the selected molecular clusters and actively promotes the ICD and the essential immuno-related processes [31]. Maintaining the temperature compensates for the energy losses, so it needs a smaller dose. The unchanged temperature with lower current density produces significantly less apoptosis as the active heating period raises the temperature [259]; Figure 10. The amount of apoptosis increases by the synergy of the temperature dynamics and the electric field, but practically does not change when the temperature stabilizes and remains approximately constant. Stochastic explanation describes this phenomenon [31]. This complexity involves the similarity of the temperature and the electric field to improve the chemical reaction rate [102]. This effect provides a possibility to improve the heterogenic selective cell destruction by mEHT in clinical practices. The therapy needs a protocol that keeps the temperature development’s dynamism [31]. Step-up heating considering the blood flow washing time (approximately 6 min) works approximately well.

Contrary to the homeostatic balancing, intensive cooling supports the growth of the incident power. Forced intensive cooling increases the current density because the incident power must increase quadratically, replacing the power taken by the cooling. Due to the applied cooling (energy loose), significantly modifying the incident power does not provide accurate dose measurement. The dose needs other direct registering, like temperature or current density j. The j flows through the patient practically independent from the energy losses, characterizes the absorbed SAR only. Consequently, the direct measurement of the current density appears as the dose in an intensive cooling process instead of the power.

**Figure 11:**
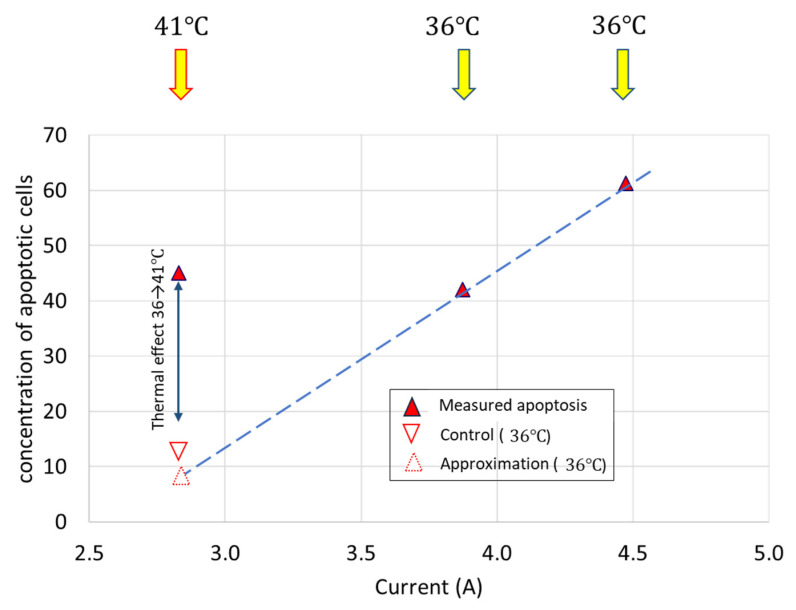
The apoptosis linearly increases by the increase of current density. The higher current density was reached by intensive cooling of the sample, keeping the medium at 36 °C, while the standard treatment was at 41 °C. The difference in the approximated apoptosis at low current at 36 and at 41 °C is produced by the thermal effect.

The apoptosis of malignant cells shows the efficacy of mEHT therapy. The apoptotic cellular degradation could be used for dosing in the active heating period [259]; Figure 11. Consequently, the connection of the apoptotic cell degradation and current density appears like an essential task of the new dose when the ***j*** is enhanced by cooling.

The current density is proportional to the percentage of apoptosis. Measurements on the U937 cell line well prove this concept [136]. The concentration of apoptotic cells grows linearly with the current density ***j*** of mEHT; Figure 11. The standard mEHT treatment was performed at 41 °C, with a standard current density. The control is a sham experiment, which fits a linear line. The heat effect of the standard treatment could be approximated from this experiment.

The current density j appears as an optimal dose of mEHT. On the other hand, the ***j*** does not offer a dose solution for conventional LRHT methods, where the patient impedance matching is far from the resonance. The measured current density in LRHT does not show the effective targeting of the tumor, having reflected imaginary parts and various other impedance losses. Temperature measurement remains mandatory in the conventional homogeneous mass heating of LRHT.

The percentage of the apoptotic processes induced by mEHT grows by increasing current density, which participates in both fundamental processes of this method: in the thermal and nonthermal action components. The thermal effects ensure the conditions for optimal nonterminal (excitation) processes and the rates of chemical reactions (mostly enzymatic assistances) afterward. We may regard the current density as a treatment dose, having the same role in mEHT as the ionizing isodose in RT.

The j represents an isodose distribution in the target with mEHT, like the beam isodose in the RT method. Note that this dose could happen only when the energy loss is low, and the overall energy intake is not as high as the heterogeneity differences that may appear with massive heating. Hence, the sensing heterogeneity limits the incident power. When the heating forces isothermal conditions, the $SAR\sim j^{2}$ dominates, and the heterogenic structure becomes thermally homogeneous. The isothermal temperature overshadows the electrical differences in the target. The electromagnetic differences become gradually visible when the incoming energy decreases. The electromagnetic effects distinguish the electrical differences when its average absorption intensity does not exceed the distinct energy levels of the difference between the absorption values of the desired differentiable units, so when the $j\geq j^{2}$. So, in conditions when j≤1, the selection of tumor cells is effective.

**Figure 12:**
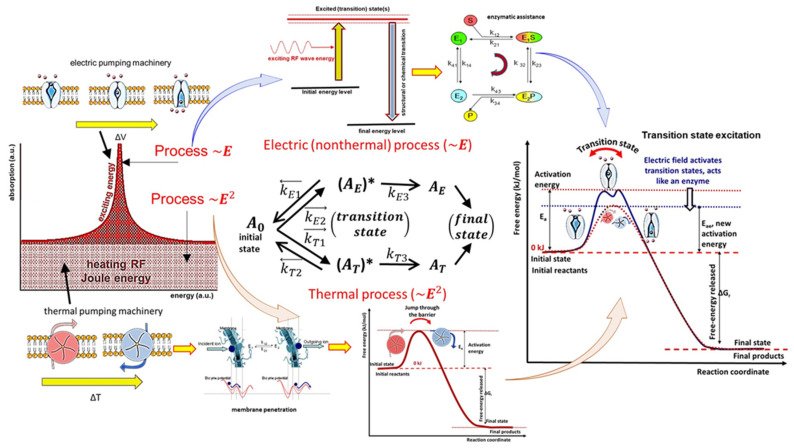
The measured thermal and nonthermal effects of mEHT. The thermal effect has an Arrhenius character, while the nonthermal effects are quantum-mechanical, promoting enzymatic processes, pushing through the transitional state. The nonthermal processes use the thermal conditions for optimal reaction rates. (For details, see in the text.). The * denotes metastable transitional state.

The proper modulated signal may trigger resonant excitations of the proteins [111], which initiates extrinsic signal pathways for apoptosis [101,253] in a dose-dependent way [259]. Consequently, the thermal factor generating hyperthermia temperatures creates an appropriate condition for the nonthermal electric field effect by optimizing the reaction rates and enzymatic reactions. The direct thermal and nonthermal effects complete each other, creating a complex synergy of mEHT actions; Figure 12.

### 4.4. Complementary to Radiotherapy

The temperature distribution in the hyperthermic process also has complex balancing. The homogeneously high temperatures (>42 °C) in LRHT could block the enzyme activity [260] and so arrest the DNA-repairing enzymes and optimize the cellular degradation of malignant cells [261]. However, they produce massive necrosis, which makes the DAMP release unstable, as well as the high temperature (>40 °C) blocking the immune-cell activity [96], which would be necessary for APC production to form tumor-specific processes. The heterogenic heating of mEHT unites the advantages of the high cellular temperature with the mild average. The thermal component of mEHT ($T_{mEHT}$) produces a mild hyperthermic average ($38\;℃\leq T_{mEHT}<40\;℃$), which is enough for a blood-flow increase [132] to sensitize the RT, but less than the immune-cell inactivation limit [96]. The temperature of the selected cells ($T_{cell}$) is well over the average ($T_{cell}\gg T_{mEHT}$), at least by 3 °C as obtained from the apoptotic rate [95,133] and tumor degradation [135,150] (see Figure 9).

Complex balancing appears in various features of the hyperthermia processes. LRHT accelerates the distortions of malignant cells, reducing the α/β ratio in the linear-quadratic model (LQM) of cell-survival in RT [262]. The LQM neglects the third term of the Taylor expansion of the function of dose (f(D)) in an exponential dependence from the efficacy ($RT_{eff}$), which is reciprocal with the cellular survival ($S_{cell}=\frac{1}{RT_{eff}}$), supporting $S_{cell}=e^{-f\left(D\right)}≅e^{-\alpha D-\beta D^{2}}$. High efficacy means a quick decrease of the $S_{cell}$ by the applied RT dose, so the quadratic term is expected to be high. The hypo- or hyper-fractionating tries to fit the α/β ratio to the survival of cellular variants [263].

It is predicted that LRHT optimizes the α/β ratio [264], which can be used for quantitative reference for an equivalent radiation dose of mEHT [129]. Due to the LRHT effect varying by types of cancer cells, the quantitative dose reference was measured on two different lung cancer cell lines, A549 and NCI-H1299. The dose escalation by mEHT well fits LQM and made it possible to estimate the reference dose determined by equivalence.

The daily RT fractions destabilize the cellular membrane [265], which is a possible general target for cancer therapy [266]. The mEHT attacks the membrane by thermal and electric field load, supporting the membrane destabilization. The double stress of mEHT (heat and field) probably also destabilizes the plasma membrane. The observed intensive apoptosis in many mEHT measurements in various tumors and the synergy with fractional RT concludes that the membrane destabilization helps the apoptosis and does not lead to necrotic cell death. The tripling of the apoptotic bodies in radioresistant pancreas tumors in mono-mEHT and mEHT + RT combined therapies [134] supports the idea that the destabilized membrane helps form apoptotic bodies.

Both the RT and the mEHT induce reactive oxygen species (ROS) as well as damaging subcellular structures and organelles (such as the cytoplasmic membrane, endoplasmic reticulum (ER), ribosome, mitochondria, and lysosome), affecting various biological activities globally altering the living processes of cancer cells, and possibly promoting autophagy too [61]. Results show the intensive promotion of autophagy with mEHT and mEHT + RT to produce apoptosis [158].

The synergy has been proven clinically in the combination of mEHT compared to RT or ChRT alone [116,179]. The frequency of LRHT and the timing with RT are essential considerations in the clinical practice of complementary therapy. The combined application of these methods has synergy, considering the complex regulations connected with both parts. The central focus of the RT makes a single or double break of a DNA strand (SSB or DSB). Inhibiting the DNA repair is the expected primary support from LRHT. RT needs radiosensitive conditions to fulfill its task, while LRHT (as shown with mEHT too [132]) gives oxygenation for the inhibition of the repair and/or arrests the activity of repair enzymes. The γH2AX monitors the repair after RT is connected to the DSB of DNA.

### 4.5. Sequences and Timing of Treatments in Complementary Therapy

Both therapies, mEHT and RT, could cause cellular destruction in their stand-alone application, inducing necrosis. mEHT in monotherapy produces massive apoptosis [134,142,150], even in radioresistant cases [148]. These distortion mechanisms are mostly independent of the subsequent therapy, while in the application as the second in the sequence, a strong dependence could be formed.

The optimal timing between RT and mEHT has a spatiotemporal complexity, challenging the sequencing and frequency of the combination. The RT defines the application sequence:

- When the oxygenation (blood flow intensity) is high, we expect sensitivity for RT, so apply it first. The maximal frequency of mEHT is every second day.
- When the tumor has hypoxic conditions (low oxygen content), apply the first mEHT to increase it and sensitize the RT.

Further considerations can modify the above sequences depending on the tumor and its grade. The temperature effect also modifies the clinical issues, so we list some features in general for HT effects, where mEHT could also be involved.

- When HT is applied first, it sensitizes the RT by oxygenation of the tumor, but there could also be an inhibitory effect when HT induces hypoxic conditions, which may happen at higher temperatures than 43 °C, which usually does not happen with mEHT.
- Both HT and RT produce heat shock proteins (HSPs). The RT-induced stress also produces these chaperone proteins in different amounts and types. For example, HSP70 and HSP27 are involved in regulating the base excision repair (BER) enzymes in response to RT stress [267].
- Developing an antiapoptotic HSP70 chaperone defines the minimal time between the repeated HT treatments. Due to the HSP70 back to the baseline 48 h post-treatment. Consequently, every second day is recommended as the most frequent application. The maximal time between the HT treatments is one week when the possible buildup of the adaptive immune system finishes.
- HT has effects that are not dependent on enzyme activity, such as a variety of irreparable DNA mismatches, heat-activated methylation, hydrolysis, mono- or di-adduct damages, etc. The activity of repairing enzymes grows by temperatures, but at high temperatures (generally 43 °C) it blocks their activity. The enzyme block could be helpful. The high temperature causes intense hypoxia in the tumor and suppresses the RT efficacy, so mild heating of mEHT is optimal.
- HT at lower temperatures is sufficient to enhance perfusion [70] and the formation of numerous reactive oxygen species (ROS), such as hydrogen peroxide, superoxide anions, nitric oxide, hydroxyl radical, etc. Superoxide dismutase (SOD) forms an essential component in the defense against ROS. Heat stress could cause a decrease in SOD levels, which also leads to cell death [268].
- There is a risk that HT could support the activity of DNA repairing enzymes when it is applied after RT, even also when the end temperature is as high to block the enzymatic activity, because the first part of the heating is a “warming up”, presenting a preheating, which could increase the activity of reparation enzymes [269].

The DSBs are typically repaired within two to six hours following RT. A higher rate of the γH2AX expression was observed at three hours as compared to one hour post-RT treatment, signaling that the DSBs are still left unrepaired [270] 3 h posttreatment. However, this could depend on the type of malignant cells [271]. By 6 h posttreatment, γH2AX decreases approximately to half the amount [272]. Combining LRHT with 2 Gy radiation, the concentration of γH2AX after 1 h at 42 °C is higher than at 39 ℃ [273], and it is observed that a shorter time between the treatment parts results in a higher number of γH2AX.

A 90 min timing between LRHT and RT significantly decreases the treatment efficacy in clinical practice compared to a shorter (60 min) delay [274]. The subsequent in vitro modeling on SiHa and HeLa cell lines [275] did not significantly impact the time interval as in the clinical data, while earlier in vitro studies showed a significant difference preferring the treatments to follow each other quickly [276]. Another in vitro experiment supports quick sequences, observing that the DSB of DNA, measured with γH2AX, vanishes after 2 h of RT [274]. Earlier, it was shown that simultaneous application has the highest efficacy [277].

A high number of patients was studied, and a large impact of timing between LRHT and RT of 4 h was not observed [278]. This contradictory result started an intensive debate between the research groups [279,280]. The discussed disagreement of the two clinical studies is confusing indeed. The reasons could have multiple components. The different devices, the sequence order of the treatments, and the frequency of the LRHT application could represent differences between the therapies and lead to a contradictory conclusion. The first thirty minutes of “warming up” could be considered preheating, which could increase the activity of reparation enzymes, including a risk that LRHT increases the DNA-repairing enzyme activity and supports the repair of DNA when LRHT is applied second in the sequence [269]. The warming-up period is mostly technically dependent, but depends on the nonlinear physiologic control of the complex regulation of the patient, which could rely on the bolus cooling and other device-dependent conditions. The warming-up period with the non-homogeneous thermal effect by mEHT behaves oppositely than conventional LRHT. mEHT generates the most significant apoptotic activity in the warming-up period [259]. When the LRHT-induced temperature is high enough (>42.5 °C), it could imply the blocking of the repairing enzymes. However, the necrotic cell-killing is also intensive in this high-temperature regime so that the DNA damage could have secondary importance in cellular degradation.

Note, the murine models in vivo (C3H mammary carcinoma) [281] show the thermal enhancement ratio (TER) extensively decreases and at the end vanishes after 4 h in both sequences when the LRHT precedes or follows RT, while the tumor control has a much narrower (30 min) and non-symmetric interval.

The cell-cycle arrest is connected to the electric field activity and is primarily non-thermal [282]. A part of the electric field penetrates the cell through the voltage-sensitive phosphatase (VSP) [283] and modifies the cytoskeletal polymerization [138]. The field-controlled phosphorous hydrolysis could have an essential role in cytoskeleton restructuring and resonant-type behavior phenomena. The amplitude-modulated carrier frequency can produce stochastic resonance, selectively inducing biological enzymatic reactions and polymerization [111].

With care about the physiologic complexity, mEHT takes this contradictory situation seriously and defines the clinical guideline for the complementary therapy, considering the BF as the primary factor [284]. When the BF is low, the RT efficacy is suboptimal; the guideline proposes applying mEHT first, increasing the oxygenation, and helping the set of RT reactions be more effective with the higher reaction rate of molecular changes promoting the fixing of the strand break in the DNA. The mild hyperthermic factor of mEHT optimizes the blood-perfusion to support the RT, and the most optimal frequency of mEHT is every two to three days [285], which well correlates with the timing relaxation of the induced protective HSP70 in the heated malignant cells [253]. This frequency of mEHT treatment fits well with the clinical evaluations, which are fixed in the internationally accepted guideline of mEHT therapy [284].

When LRHT or mEHT is the first in the chosen sequence, it provides oxygenation, which sensitizes the RT and produces protecting HSPs. The RT-induced stress also produces repairing chaperone proteins, like HSP70 and HSP27, which regulate the base excision repair (BER) enzymes in response to RT stress [267]. In addition, the heat effect has other enzyme-independent effects such as sensitizing to the RT: it could cause a variety of irreparable DNA mismatches, heat-activated methylation, hydrolysis, etc.

Mild heating also produces a sufficient enhancement of blood perfusion [70] and enhances the formation of numerous reactive oxygen species (ROS), such as hydrogen peroxide, superoxide anions, nitric oxide, hydroxyl radicals, etc. The heat stress could decrease the superoxide dismutase (SOD) level, weakening the defense against ROS, leading to cell death [268]. mEHT increases the ROS level more extensively than homogeneous (isothermal) heating [136], supporting the RT. Other physiological effects of heating (such as the increase in the electrolyte transport systems like the blood flow and lymph) could enhance the success of RT, together with the increased oxygenation. However, there could also be an inhibitory effect when LRHT induces hypoxic conditions, which may happen at higher temperatures, while mEHT reduces the hypoxic level [286], vastly promoting the better efficacy of RT.

### 4.6. Immunogenetic Effects

The heat and electrical stresses produce HSP chaperone proteins with mEHT to protect the cells from stress damage. The most characteristic protein family of chaperones, HSP70, acts like a “double edge sword” [287,288], exhibiting both inflammatory and anti-inflammatory, protumoral or antitumoral, immune stimulator or immune suppressor, etc. functions. The role of HSPs depends on the conditions of their activity forming “friends or foes” [289,290,291]. The primary function of intracellular HSPs (iHSPs) is to avoid the cell’s apoptosis and protect the cell’s living conditions irrespective of its malignant or healthy state. Nevertheless, certain conditions may promote the secretion of HSPs in the transmembrane position (mHSPs) or their escape extracellularly to the TME milieu (eHSPs). mHSPs may signal to make malignant cells recognizable to NK cells [169]. eHSPs could offer even more help in the elimination of malignancies. The mHSP70 carries an “info signal” [292], with the genetic properties for producing antigen-presenting cells (APCs) and creating killer T-cells [293], by the maturation of dendritic cells (DCs) [294]. This process requires that the destruction of the cell is “gentle enough” and does not degrade the DAMP proteins. When the appropriate molecules have a particular spatiotemporal order (immunogenic cell death, ICD), the set of molecules ensures that the mHSP70 becomes a forceful “friend” losing its “double-edge sword” behavior, and the genetic info well maturates the DCs forming APCs. The process directly applies immune-oncology principles, and so ICD is of tremendous clinical interest [295].

The major achievement of mEHT is activating the innate and adaptive immune system to eliminate tumor cells both locally and systemically in the whole body. The induced mHSPs mark cancer such as to be recognized by the innate immune action with NK cells [169]. The secretion of eHSPs and the correct spatiotemporal set of DAMP may develop tumor-specific adaptive immune processes to attack the cancer cells all over the body.

In such a way, mEHT turns the local treatment systemic (abscopal), as proven preclinically [170,174] and clinically too [116,179,296].

The abscopal effect was discovered in RT more than 60 years ago [297], but its application was hindered because it was observable only in low radiation doses, limiting the expected direct local degradation. The recent rediscovering of the abscopal effect with RT shifts the idea from myth to reality [298] and sees it explained by molecular processes [299]. The synergy of RT with the emerging checkpoint inhibitor and antibody immune-therapies provides new curative possibilities [300,301,302]. This field could have a new combination: mEHT supported TSI develops immune adaptation by the tumor antigens providing an abscopal addition to local RT.

**Figure 13:**
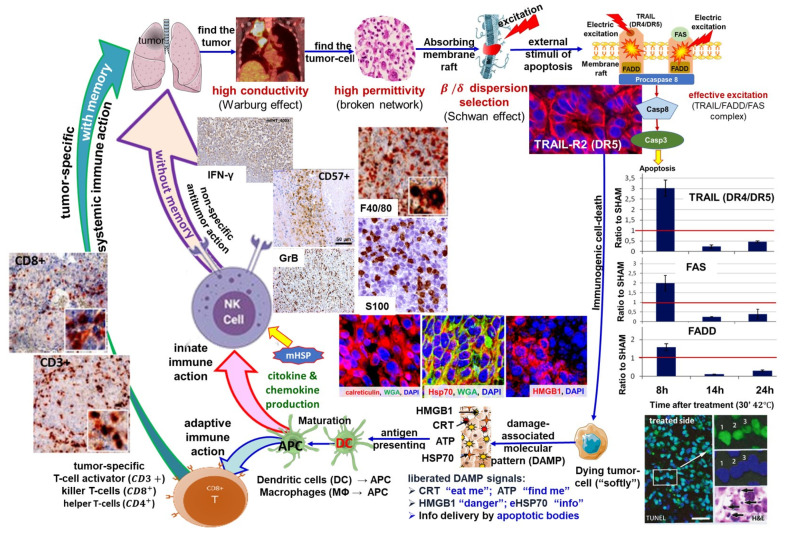
The negative feedback structure of the abscopal effect shows a complex loop from recognizing the antigens to their use in tumor-specific immune processes. The experiments are from various publications. The loop summary only demonstrates how the loop works. The measurements are from the following publications: the selection line reviewed [101], TRAIL-R2-FAS-FADD complex [153]; apoptosis [133,150], ICD [305]; DAMP [174], APC [163] immune [172], NK, Granzyme [169], IFN-γ [182], CD3+, CD8+ [163,170].

The synergy of mEHT and RT turns these local treatments systemic, creating tumor-specific immune processes (TSI) that extend the abscopal effect. The immunotherapy strategy optimizes the RT with mEHT for the best efficacy [303] and highest safety [178]. The abscopal effect could renew the complementary applications of RT with this theranostic synergy and well fits to the emerging trend of immuno-oncology too. This function connects mEHT to the emerging trend in the field, to immuno-oncology [304]. The in-situ feedback loop of the immune effects of mEHT is shown in Figure 13.

**Figure 14:**
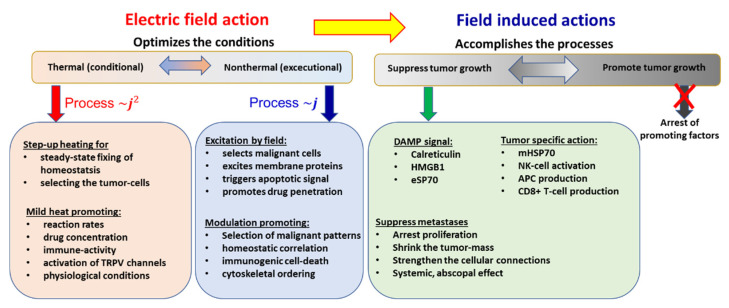
The processes of thermal and nonthermal effects of selective, heterogenic heating. The field-induced actions are complex, requiring both the thermal (conditional) and nonthermal (excitation) processes.

Finally, we may conclude that the thermal and nonthermal effects represent the nonlinear ($\sim j^{2}$) and linear (~j) dependence of the current density and in consequence of the electric field, but their functions differ. The thermal effect ensures the general energy background, while the nonthermal is resonant; Figure 14.

The synergy of mEHT with radiotherapy completes the advantages with essential factors additionally to the conventional heating processes; Table 3.

## 5. Summary

To solve the challenges of conventional LRHT, mEHT has modified the isothermal concept of oncological hyperthermia, focusing on the cellular distortion of malignant cells. The new paradigm strongly considers the goal of LRHT, concentrates on the malignant cells, and destroys them in the targeted volume. The principal idea is to use the natural heterogeneity of the cancerous tissue, using the particular living conditions of malignant cells, making them different from healthy cells and healthy host tissue. mEHT has an isodose. The RF current density is defined similarly to the ionizing isodose in RT practice. The degradation of the malignant cells and controllable stable dosing guides the efforts in synergy with RT.

Modulated electro-hyperthermia complements radiotherapy with the precise heterogenic cellular selection of malignant cells. The transmembrane protein clusters (rafts) are excited by mEHT and heated in synergy with the double-strand breaking of the DNA by RT. The synergistic harmony of ionizing, thermal, and nonthermal effects allows the immunogenic cell death of the malignant cells and develops tumor-specific immune actions in both the innate and adaptive immune system in situ during the treatment. The recognition characteristic is amalgamated with the curative therapy, so the mEHT + RT synergy is theranostic.

The selection process of mEHT uses the malignant attributes that characterize all malignancies: the metabolic, dynamic, and structural differences. This universality of mEHT does not depend on the mutation variants of cancer. Consequently, mEHT—like RT—independently breaks the DNA strands of various malignant mutants, so the synergy of the two methods may form a forceful cancer therapy. The final result is a systemic (abscopal) effect that destroys the malignant cells in the entire body irrespective of the possibility of its visual imaging. The complex integrating effect of mEHT + RT triggers physiologic and cellular changes by thermal and ionizing components. Additionally, the complementary application to RT triggers molecular and immunological changes with resonant and ionizing excitation. All complex balances have progenitors of functioning promoters and suppressors for balancing.

**Figure 15:**
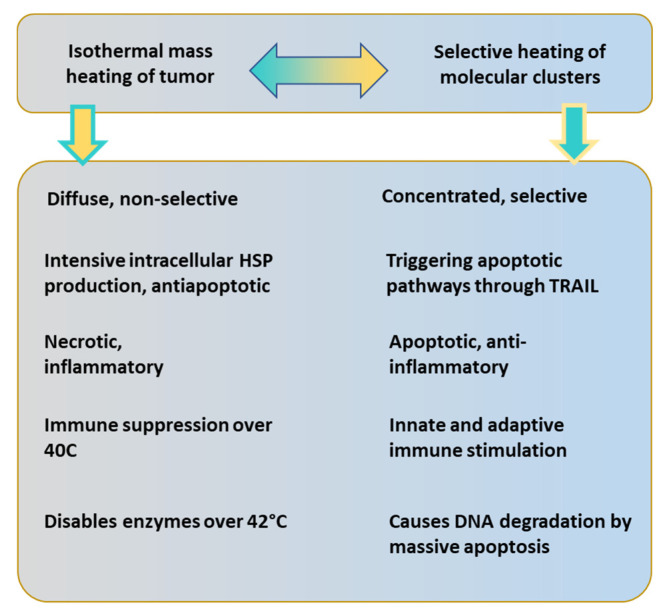
The major differences between isothermal and selective paradigm heating are listed in the columns.

mEHT changes the LRHT paradigm from homogeneous mass heating to a heterogeneous selective one. The difference between the two approaches has been proven in various experiments. Figure 15 shows a rough comparison of mass heating with selective heating.

## 6. Conclusions

mEHT results well prove the nanothermia efficacy and its conceptual success. The synergy with RT delivers effective cell degradation in tumors and develops an abscopal effect, using the homeostatic adaptation of the healthy immune regulation to degrade the malignant cells systemically in the entire body. The synergy is verified by preclinical and validated by clinical results.

## Figures and Tables

**Table 1:** The synergistic possibility shows a broad range of advantages for combined therapy of LRHT and RT.

Tumor Characteristics	Oncological Hyperthermia Including All Technical Solutions	Synergy with Radiotherapy
**Cell cycle**	Arrests the cycle of cells at the S stage, activates the malignant cell from its dormant (G0) phase making attack possible for chemo- and radio-therapies	Radiotherapy arrests the M/G2 stages of the cell cycle well completes the arrest
**pH dependence**	Kills cancer cells in an acidic environment (Hippocrates’ original idea)	It kills cancer cells in an alkaline environment, completes the cell desertion in all environmental conditions
**Oxygenation**	Acts in the hypoxic state	Acts in an oxygenated state
**Increased temperature**	Heated tumor mass increases the oxygen delivery	Makes strand breaks on DNA, the fixing of which means oxygen blocks the reparation

**Table 3:** The essential addition of mEHT to the synergistic RT-with-hyperthermia methods.

Synergistic Addition of Modulated Electrohyperthermia	
**Nanoscopic action**	Selects malignant cells and nonthermally excites, marginal heating of the healthy cells renders less vulnerable to ionizing radiation
**Apoptotic effect**	Mostly natural apoptosis, no inflammation, no large cytokine liberation, no extra injury current, no extra pH hypoxia
**Immune effect**	Immunogenic processes, abscopal effect. Both the innate and adaptive immune system are activated, vaccination facility (patented)
**Homeostatic effect**	Harmonized with homeostatic controls, the temperature increase in the nuclei is moderate, does not make an additional enzymatic activity for reparation
**Side effects**	Lower incident power puts less load on the skin, which is anyway irritated by radiotherapy, so the synergy has fewer adverse effects
**Quality of life**	Improves quality of life by reducing side effects
**The broad range of application**	Possible to combine with radiotherapy in localizations which were not possible with radiative hyperthermia (like the brain)
**Applicable for palliative conditions**	Resensitizes to radiotherapy in highly metastatic advanced refractory cases, when conventional therapies are ineffective
**Long-time application**	mEHT is applicable as a chronic treatment for as long as is necessary with radiotherapy complementation
**Applicability**	mEHT is applicable with most comorbidities as well as in combination with any other oncotherapies
